# A Small Molecule Reacts with the p53 Somatic Mutant Y220C to Rescue Wild-type Thermal Stability

**DOI:** 10.1158/2159-8290.CD-22-0381

**Published:** 2022-10-05

**Authors:** Keelan Z. Guiley, Kevan M. Shokat

**Affiliations:** 1 Department of Cellular and Molecular Pharmacology and Howard Hughes Medical Institute, University of California, San Francisco, San Francisco, California.

## Abstract

The transcription factor and tumor suppressor protein p53 is the most frequently mutated and inactivated gene in cancer. Mutations in p53 result in deregulated cell proliferation and genomic instability, both hallmarks of cancer. There are currently no therapies available that directly target mutant p53 to rescue wild-type function. In this study, we identify covalent compsounds that selectively react with the p53 somatic mutant cysteine Y220C and restore wild-type thermal stability.

**Significance:**

The tumor suppressor p53 is the most mutated gene in cancer, and yet no therapeutics to date directly target the mutated protein to rescue wild-type function. In this study, we identify the first allele-specific compound that selectively reacts with the cysteine p53 Y220C to rescue wild-type thermal stability and gene activation.

*See related commentary by Lane and Verma, p. 14.*

*This article is highlighted in the In This Issue feature, p. 1*

## Teaser

An allele-specific covalent small molecule was identified that selectively reacts with the mutant cysteine Y220C of p53 and leads to the rescue of wild-type thermal stability levels.

## INTRODUCTION

The most commonly mutated gene in cancer is the transcription factor and tumor suppressor *TP53* (p53). Unlike *RB1*, *CDKN2A*, and *PTEN*, which are lost in tumors through homozygous deletion, *TP53* is most frequently found with a somatic missense mutation in either a heterozygous setting or with a 17p deletion and loss of the second allele (1). A germline mutation in one *TP53* allele results in Li-Fraumeni syndrome, a disorder that increases the risk of cancer occurrence by 70% to 100% over an individual's lifetime (2). Cells carrying a *TP53* mutation accumulate high levels of mutant p53 protein, which drives a dominant-negative effect on the wild-type (WT) copy and the p53 homologs p63 and p73 (3). The primary transcriptional targets of p53 WT are p21 (*CDKN1A*), which functions as a potent cell-cycle inhibitor, MDM2 (*MDM2*), the E3 ligase for p53, and proapoptotic Bcl-2 family proteins (*BBC3, BAX*, and *NOXA*). Genetically engineered mouse models have shown that restoration of p53 WT activity in p53-deficient cancers promotes tumor regression and a cure (4, 5). The abundance of mutated p53 in tumors highlights the therapeutic potential of a small molecule capable of reverting mutant p53 to its WT form.

The most frequent p53 missense mutations in cancer are found within the DNA-binding domain (DBD; Supplementary Fig. S1A and S1B), where all mutations result in lower target gene expression through the loss of DNA-binding affinity (6). Structural characterization of these hotspot mutations revealed a druggable cavity in p53 Y220C (7), a mutation that indirectly inhibits DNA binding through the loss of thermal stability in the DBD (Supplementary Fig. S1C). The small-molecule PhiKan083 (8) was subsequently developed to bind within the p53 Y220C cavity and has undergone several chemical iterations to improve both affinity and thermal stabilization (9). Although the PhiKan compounds have demonstrated the potential targeting of p53 Y220C, none have reached biochemical potency or showed thermal stabilization to WT levels, and thus are unlikely in their current form to satisfy the requirements of drug candidates.

The discovery (10) and recent FDA approval of the cysteine-reactive KRAS G12C inhibitor sotorasib (11) demonstrates the therapeutic potential of covalently targeting somatic mutant cysteines in proteins that lack canonical ligand-binding pockets. The weak reversible affinities of KRAS G12C inhibitors were overcome by the kinetics of the acrylamide-cysteine Michael addition reaction to achieve high biochemical potency (12). In addition, several small-molecule correctors of protein misfolding have also recently gained FDA approval for treating cystic fibrosis (CFTR), Fabry disease (αGAL A), transthyetin amyloidosis (TTR), and sickle cell disease (HbS; ref. 13).

In this study, we identified the first covalent compounds that selectively react with the somatic cysteine in p53 Y220C to restore thermal stability to WT levels. Moreover, our structural analysis revealed a novel conformational state of the p53 DBD that serves as a potential druggable pocket for future studies. The findings and compounds presented herein represent a therapeutic strategy to treat over 125,000 cancer patients annually carrying the p53 Y220C mutation (9) and as a prophylactic therapy to lower the cancer risk in Li-Fraumeni syndrome.

## RESULTS

### Covalent Carbazoles Labeled but Minimally Stabilized p53 Y220C

**Figure 1.**
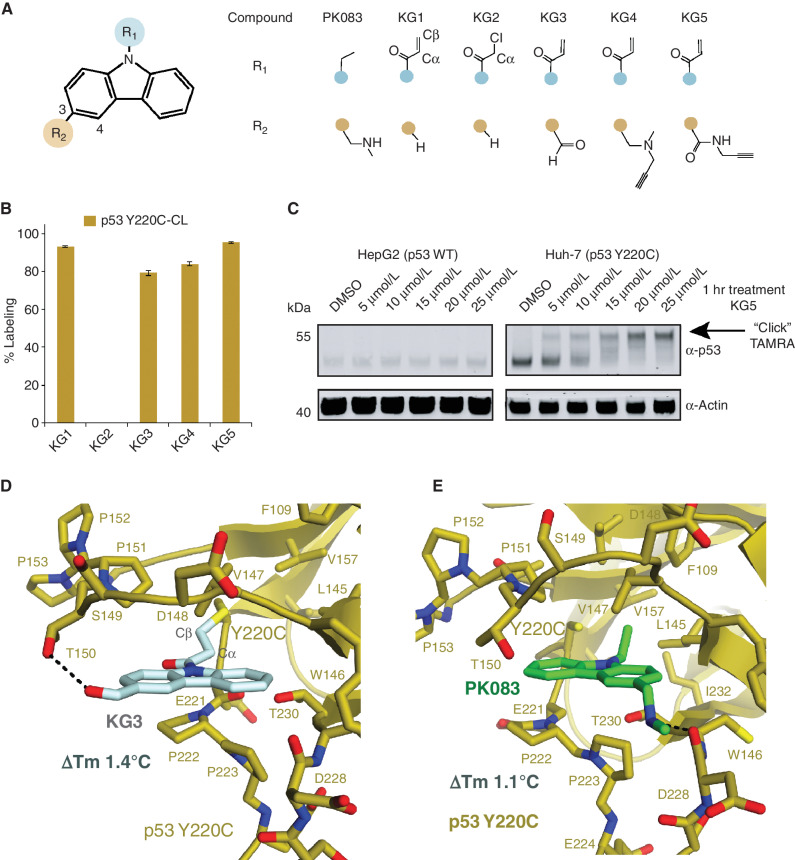
Development of a covalent PhiKan083. **A,** Chemical structures of the carbazole series. **B,** 1 μmol/L p53 Y220C-CL was incubated with 10 μmol/L of the carbazole compounds at 4°C for 24 hours. Adduct formation was observed by LC/MS. **C,** HepG2 (p53^WT/+^) or Huh-7 (p53 ^Y220C/−^) cells were treated with the indicated KG5 concentration for 1 hour at 37°C and target engagement was observed by gel shift following a copper click reaction with TAMRA-azide. **D,** Crystal structure of p53 Y220C-CL bound to KG3 at 2.4 Å resolution. **E,** Crystal structure of the p53 Y220C–PhiKan083 complex (Protein Data Bank 2VUK; ref. 8).

To generate a covalent p53 Y220C stabilizing compound, we began with a carbazole scaffold and attached an acrylamide (KG1) or chloroacetamide (KG2) electrophile to the carbazole nitrogen (Fig. 1A). We hypothesized that these electrophiles would be positioned in the Y220C cavity similar to the ethyl group in PhiKan083 (ref. 8; Fig. 1A) and would react directly with the mutant cysteine 220. To test Y220C-specific covalent labeling, we designed a cysteine light p53 Y220C DBD (Y220C-CL; Supplementary Fig. S1D) by mutating the surface cysteines C124S, C182S, C229S, C275S, and C277S. Notably, mild alkylating compounds have been found to react with C124, C182, and C277 (14, 15), but not Y220C.

We incubated 10 μmol/L KG1 with 1 μmol/L p53 Y220C-CL at 4°C for 24 hours and found significant KG1 labeling (92.9% ± 0.4%) and no KG2 labeling (Fig. 1B). Cysteine light p53 WT (p53 WT-CL) showed no labeling for either compound, indicating the reaction is specific to the Y220C cysteine. The inherent reactivity of chloroacetamide is greater than acrylamide toward thiols (16), suggesting that the acrylamide Cβ in KG1 is better positioned for the C220 nucleophilic attack compared with the Cα in the chloroacetamide KG2 (Fig. 1A).

To determine the stabilizing effect of KG1, we first measured the melting temperature (Tm) for p53 Y220C-CL (33.21°C ± 0.02°C) and p53 WT-CL (41.59°C ± 0.02°C) alone and calculated the change in Tm (ΔTm) for the Y220C mutation to be −8.40°C ± 0.02°C (Supplementary Fig. S2A). We then fully labeled p53 Y220C-CL with KG1 and purified the complex by gel filtration to remove excess compound. The ΔTm for KG1 was determined to be +1.28°C ± 0.01°C, similar to the PhiKan083 ΔTm of +1.11°C ± 0.06°C at 250 μmol/L (Supplementary Fig. S2A).

In an effort to improve the covalent carbazole interaction with p53 Y220C, we installed H-bond donating groups at the 3-position (Fig. 1A) to establish an H-bond interaction with the D228 backbone carbonyl (8). The amide KG5 provided the greatest labeling (95.2% ± 0.5%) compared with the aldehyde KG3 (79.0% ± 1.0%) or methylamine KG4 (84% ± 1%; Fig. 1B; Supplementary Fig. S2A–S2D). To determine whether KG5 engages endogenous p53 Y220C, we treated HepG2 (p53^+/+^) or Huh-7 (p53^Y220C/−^) cells with 5 to 25 μmol/L KG5 for 1 hour and performed a click reaction between TAMRA-azide and KG5's alkyne in cell lysate to induce a mobility shift visible by SDS-PAGE Western blot. KG5 was found to fully engage p53 Y220C at 20 μmol/L with no engagement observed in WT (Fig. 1C).

To determine the binding mode of the covalent carbazole, we solved a cocrystal structure of p53 Y220C-CL bound to KG3 at 2.4 Å resolution (Fig. 1D; Supplementary Table S1). The overall fold of the p53 Y220C-CL is comparable with p53 Y220C–PhiKan083, with a root-mean-square deviation (RMSD) of 0.383 Å; however, there are several differences in the small molecule's binding mode. The 3-positions of the two carbazoles face opposing sides of the p53 Y220C cavity, where the aldehyde on KG3 forms an H-bond with T150, whereas the 3-position methylamine on PhiKan083 forms an H-bond with the carbonyl backbone of D228 (Fig. 1D and E). The covalent KG3–C220 bond orients the acrylamide Cα and Cβ outside of the Y220C hydrophobic cavity between C220 and L145, resulting in the loss of the van der Waals (vdW) interactions with p53 V147, L145, F109, L257, and V157, which were observed with the ethyl group in PhiKan083 (Fig. 1E) or pyrrole in other reversible compounds (17). Interestingly, it was originally predicted through computational docking that PhiKan083 would adopt the same conformation that we observed for KG3 (8).

Although our covalent carbazole series labeled recombinant and endogenous p53 Y220C, the compounds provided only minimal thermal stabilization. The p53 Y220C-KG3 crystal structure presented an opportunity to enhance stability and labeling by creating a new series of compounds. We hypothesized that positioning a methyl group on the Cα of the acrylamide would mimic the ethyl group in Phikan083 and gain vdW interactions with p53 V147, L145, F109, L257, and V157. In addition, the altered binding conformation of the carbazole ring suggested that installing H-bond donors on the 4-position would establish interactions with the D228 carbonyl (Fig. 1D).

### Methacrylamide Indole Series Enhanced Stabilization of p53 Y220C and Induced a Novel Conformation

**Figure 2.**
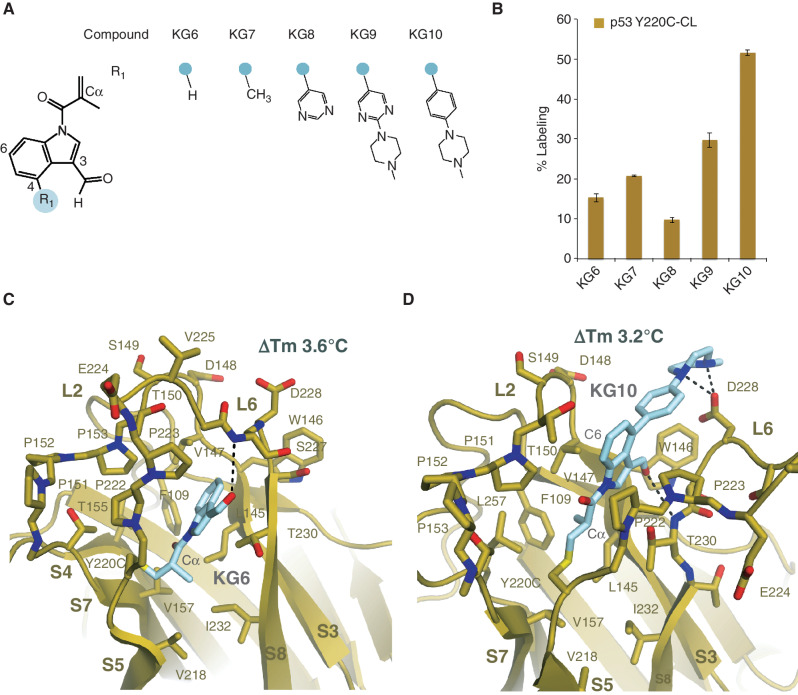
A methacrylic indole compound induced a novel p53 conformational state. **A,** Chemical structures of methacrylic indole compound series. **B,** 1 μmol/L p53 Y220C-CL was incubated with 10 μmol/L indole series at 4°C for 24 hours. Adduct formation was analyzed by LC/MS. **C,** Crystal structure of p53 Y220C-CL bound to KG6 in a novel conformational state at 1.6 Å resolution. **D,** Crystal structure of p53 Y220C-CL bound to KG10 at 2.0 Å resolution.

Insertion of a Cα methyl acrylamide onto the carbazole produced a labile amide bond to the carbazole presumably due to a steric clash with the phenyl ring of the carbazole. To avoid this clash, the carbazole scaffold was replaced with an indole (Fig. 2A). We found that the indole methacrylamide KG6 did not react as efficiently as KG3, with 15% ± 1% labeling compared with 79% ± 1%, respectively (Fig. 2B; Supplementary Fig. S3A–S3E). However, the thermal stability was enhanced with a ΔTm of +3.563°C ± 0.002°C for KG6 compared with +1.4°C ± 0.1°C for KG3. Indeed, a Cα methyl substitution on an acrylamide is known to substantially lower the reactivity toward thiols due to the steric hindrance of the nucleophile (18). We next tested substitutions at the 4-position of the indole to enhance labeling efficiency through ionic interactions with the p53 D228 carbonyl (Fig. 2A). The phenyl–piperazine compound KG10 was found to have improved labeling (51.6% ± 0.7%) while maintaining a significant shift in thermal stability (ΔTm +3.21°C ± 0.03°C).

To determine the structural mechanism of stabilization by the methacrylamide indole series, we solved cocrystal structures of p53 Y220C-CL in complex with KG6 or KG10 at 1.6 Å and 2.0 Å, respectively (Supplementary Table S1 and Supplementary Fig. S4A–S4D). Strikingly, the p53 Y220C–KG6 complex shows significant structural changes in L6, where the Cα position of E224 is altered by 13.2 Å (Fig. 2C) relative to p53 Y220C-CL–KG3, whereas the overall fold remains similar (RMSD of 0.454 Å). This closed conformation of L6 in the p53 Y220C–KG6 complex has not been observed in previous p53 crystal structures. The significant stability enhancement by KG6 is driven by establishing a hydrophobic network within the core of the immunoglobulin fold at β-strands S3, S5, S7, and S8 (Fig. 2C). vdW contacts are formed between the Cβ of the acrylamide on KG6 with p53 V157 and V218, and between the Cα methyl group of the acrylamide on KG6 with L145, V218, T230, and I232. The KG6 methyl acrylamide contacts are bridged to β-strands S3 and S8 through interactions between the indole ring and p53 T230 and V147. The alternate binding mode of KG6 compared with KG3 positions the aldehyde of KG6 to form an H-bond interaction with the backbone amine of S227 instead of T150. Finally, an allosteric stabilization effect was observed as a result of the L6 movement, where the D228 carbonyl is shifted 2.9 Å, establishing an intramolecular H-bond with the backbone amine of V147 rather than through the small molecule itself (Supplementary Fig. S4E).

Although KG6 induces a novel p53 Y220C conformation, the L6 position and overall fold in the Y220C–KG10 complex are similar to KG3, with an RMSD of 0.367 Å (Fig. 2D). The elongated KG10 molecule makes distal contacts with p53 Y220C, spanning from the core of the immunoglobulin fold to L6 D228 outside of the pocket (Fig. 2D). As predicted, the Cα methyl of the acrylamide is positioned in the C220–L145 cavity similar to the PhiKan083 ethyl (Figs. 1E and 2D), making vdW interactions with p53 F109, L145, V147, and L257. The piperazine makes H-bond interactions with D228, and a D228A mutation reduced labeling from 51.6% ± 0.7% to 37.2% ± 0.5%. No change in labeling was observed with the D228A mutation from compounds KG6 and KG7, which lack the piperazine moiety. The labeling of compound KG7 was not equivalent to KG10 with the D228A mutation, suggesting that the piperazine is also making favorable vdW contacts with p53 W146. In contrast to KG3, the KG10 3-position aldehyde is oriented into the Y220C cavity and forms an H-bond with the backbone amine of T230. However, W146 is shifted 0.8 Å away from the cavity relative to Y220C–KG3 and 1.5 Å relative to Y220C–KG6.

### Methacrylamide 6-Azaindole Rescued WT DBD Stability

**Figure 3.**
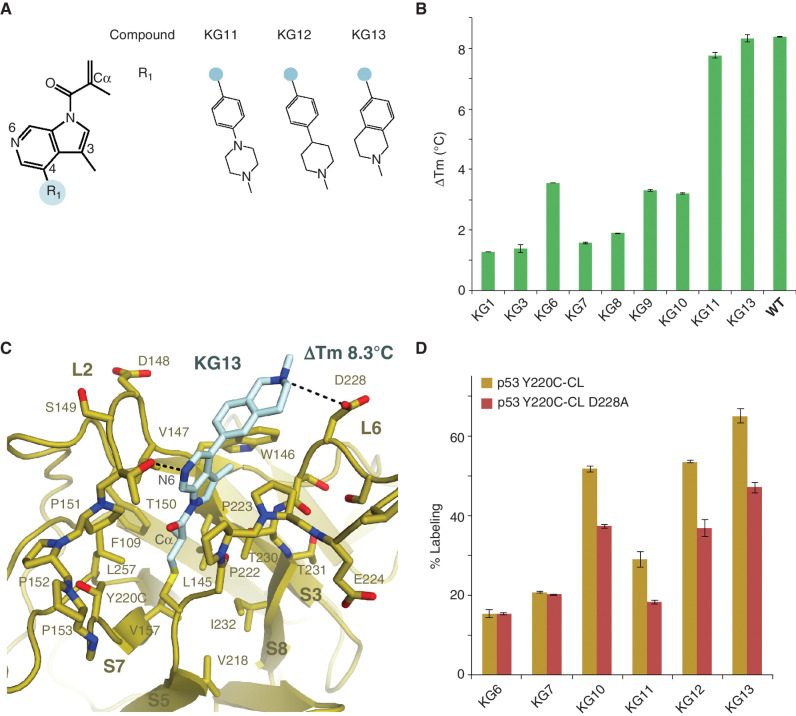
Azaindole compounds stabilized p53 Y220C to WT levels. **A,** Chemical structures of the azaindole series. **B,** p53 Y220C-CL was fully labeled by the compounds and purified by size-exclusion chromatography, and its thermal stability was analyzed by differential scanning fluorimetry. The difference in Tm relative to unliganded p53 Y220C-CL is displayed. **C,** Crystal structure of p53 Y220C-CL bound to KG13 at 1.7 Å resolution. **D,** 1 μmol/L of p53 Y220C-CL or p53 Y220C-CL D228A was incubated with 10 μmol/L compound at 4°C for 24 hours. Adduct formation was analyzed by LC/MS.

Although we predicted the aldehyde in KG10 would interact with T150, the crystal structure revealed that the 3-position was facing the hydrophobic pocket formed by V147, W146, and C229 on the opposing side of the Y220C crevice (Fig. 2D). A methyl substitution at the 3-position would be predicted to favor the hydrophobic pocket, and removal of the electron-withdrawing group from the ring would reduce the electrophilicity of the acrylamide, thereby lowering off-target engagement in cells. In addition, p53 T150 is positioned adjacent to C6 of the indole ring in the Y220C–KG10 structure (Fig. 2D), suggesting that a nitrogen substitution at C6 would further enhance stability and labeling through an H-bond interaction with T150. Strikingly, we found that these substitutions together enhanced the thermal stability of p53 Y220C near that of WT, with a ΔTm of +7.8°C ± 0.1°C (Fig. 3A and B).

Having observed a significant thermal shift after changing the core scaffold from an indole to an azaindole, we next tested the thermal stability contribution of the D228 interaction with phenyl-piperazine in KG11. The D228A mutation showed no difference in overall p53 thermal stability in the absence of ligand, with a Tm of 33.47°C ± 0.2°C for p53 Y220C-CL D228A compared with 33.21°C ± 0.02°C for p53 Y220C-CL. However, the ΔTm when bound to KG11 was +5.91°C ± 0.05°C for p53 Y220C-CL D228A compared with +7.8°C ± 0.1°C for p53 Y220C-CL, highlighting the significance of this ionic interaction for both labeling efficiency and stabilization (Fig. 3B). To further strengthen the D228 interaction, we substituted the piperazine for a stronger base, piperidine. The substitution resulted in increased labeling from 29% ± 1% for KG11 to 53.4% ± 0.3% for KG12. Finally, to make the molecule more rigid, the phenyl piperidine (KG12) was fused to produce a tetrahydroisoquinoline (KG13). The KG13 azaindole molecule labeled p53 Y220C-CL at 65% ± 2% and stabilized p53 Y220C back to WT levels with a ΔTm of +8.3°C ± 0.1°C (Fig. 3B).

To determine the structural mechanism of stabilization by the azaindole KG13, we solved a cocrystal structure of p53 Y220C-CL in complex with KG13 at 1.7 Å (Fig. 3C; Supplementary Table S1). The binding mode and overall protein fold are similar to p53 Y220C–KG10, with an RMSD of 0.095 Å. As predicted, the aldehyde to methyl substitution at the 3-position resulted in W146 orienting 2.7 Å back into the Y220C crevice, being positioned 3.5 Å from the methyl group, and making vdW contacts. The N6 substitution to the indole ring shifted the ring 0.6 Å toward the hydroxyl in T150 and established a new H-bond interaction (Fig. 3C). The Cα of D228A is shifted 1.8 Å relative to p53 Y220C–KG10, with the fused ring piperidine positioned to establish an H-bond with D228. A D228A mutation resulted in reduced KG13 labeling efficiency from 65% ± 2% for p53 Y220C-CL to 50% ± 1% for p53 Y220C-CL D228A (Fig. 3D).

We next tested whether the azaindole KG13 alters DNA-binding specificity. Using an electrophoretic mobility shift assay (EMSA), we tested *CDKN1A* promoter binding with p53 WT-CL, p53 Y220C-CL, and p53 Y220C-CL–KG13. We found the apparent Kd for *CDKN1A* to be between 2 and 3 μmol/L for all p53 WT and Y220C samples tested at 25°C, suggesting DNA specificity is not compromised following KG13 binding to p53 Y220C (Supplementary Fig. S5A–S5E). In contrast, the DNA-contact mutant R273C-CL showed negligible binding (Supplementary Fig. S5F).

### Azaindole Series Specifically Labeled p53 Y220C but Not WT Cysteines

**Figure 4.**
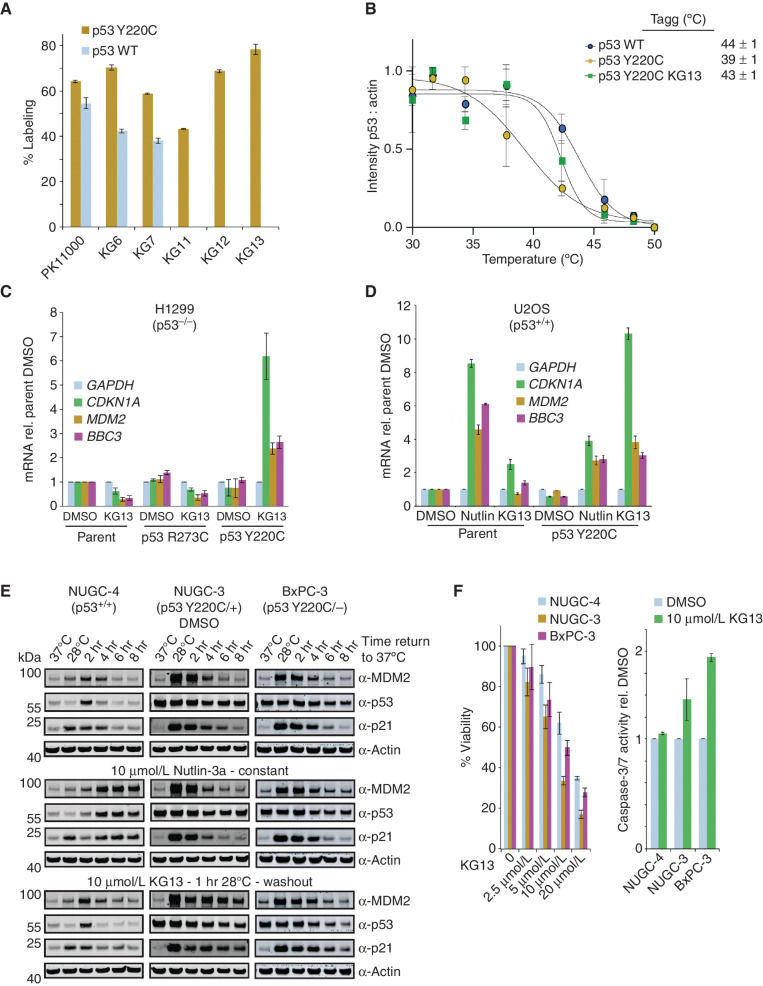
KG13 stabilized cellular p53 Y220C and activated target gene expression. **A,** 1 μmol/L of p53 WT or p53 Y220C was incubated with 10 μmol/L of compound at 4°C for 24 hours. Adduct formation was analyzed by LC/MS. **B,** CETSA intensity values were plotted with calculated Tagg values, where the KG13-treated sample showed an increase in thermal stability of cellular p53 Y220C compared with DMSO control. **C,** RT-qPCR results for 25 μmol/L KG13-treated H1299 cells stably expressing p53 R273C or Y220C. rel., relative. **D,** RT-qPCR results for 10 μmol/L Nutlin-3a (Nutlin) or 25 μmol/L KG13-treated U2OS cells stably expressing p53 Y220C. **E,** Western blot for DMSO, 10 μmol/L Nutlin-3a, and 10 μmol/L KG13-treated cell panel. **F,** Viability and caspase-3/7 activity for NUGC-4, NUGC-3, or BxPC-3 cells treated with KG13.

Our goal of generating a mutant-specific p53 Y220C–reactivating compound required selectivity toward the somatic mutant cysteine Y220C. Sulfonylpyrimidines have been shown to alkylate the p53 DBD nonspecifically, where PK11000 reacts with cysteines 182 and 277 (15). Using PK11000 as a control nonspecific alkylating compound, we tested 10 μmol/L PK11000 for 24 hours at 4°C on a noncysteine light p53 DBD with (p53 Y220C) or without (p53 WT) the Y220C mutation and observed two labeling events on both proteins (Fig. 4A). We next tested the carbazole KG5, indole scaffold KG6 or KG7, and the azaindole series KG11, KG12, and KG13 (Fig. 4A) on p53 WT and Y220C. The carbazole KG5 was found to be highly reactive with several labeling events on both p53 WT and Y220C. The indole scaffolds KG6 and KG7 also labeled both p53 WT and p53 Y220C; however, the total labeling events were reduced to two, with greater selectivity for p53 Y220C compared with PK11000 (Fig. 4A) The difference in reactivity between the carbazole KG5 and the indole KG6 or KG7 is likely attributed to reducing the electrophilicity of the acrylamide through the Cα methyl substitution. Finally, KG11, KG12, and KG13 labeled 43.2% ± 0.4%, 68.7% ± 0.7%, and 78% ± 2%, respectively, for p53 Y220C with no labeling of WT (Fig. 4A).

### Azaindole Stabilized Cellular p53 Y220C and Activated p53 Target Genes

To determine whether a Y220C-specific covalent compound could rescue p53 Y220C stability in cells, we performed a cellular thermal shift assay (CETSA) using KG13. H1299 cells (p53^−/−^) stably expressing p53 Y220C were treated with DMSO or 100 μmol/L KG13 for 1 hour, harvested, and heat treated from 30°C to 50°C before being analyzed by Western blot. Given that the p53 Y220C mutant is unstable at 37°C, we incubated the cells at 28°C for 24 hours prior to and during compound treatment to obtain the melting curve. The WT-specific conformational antibody Pab1620 (19) was able to immunoprecipitate p53 Y220C at 28°C but not 37°C, confirming proper folding of the DBD at the permissive temperature (Supplementary Fig. S6A–S6C). In the absence of a stabilizing ligand, p53 Y220C began aggregating at 37°C with an aggregation temperature (Tagg) of 39°C ± 1°C (Fig. 4B). For p53 WT, we observed no difference in protein stability between 30°C and 37°C with a Tagg 44°C ± 1°C, consistent with full-length recombinant p53 WT (20). Finally, we treated cells expressing p53 Y220C with 100 μmol/L KG13 for 1 hour and observed a rescue of stability at 37°C, and a shift in Tagg to 43°C ± 1°C, similar to p53 WT (Fig. 4B; Supplementary Fig. S6D–S6F).

After observing KG13-stabilized cellular p53 Y220C, we next monitored the compound's effect on gene expression, protein induction, and p53 Y220C promoter occupancy upon warming cells to physiologic temperature. The isogenic cell panels H1299 (p53^−/−^) and U2OS (p53^+/+^) stably expressing p53 Y220C were treated with 25 μmol/L KG13 for 1 hour at 28°C, washed with media, and transferred to 37°C for 8 hours before analysis. *CDKN1A* was the most upregulated p53 target gene by KG13, with a 7 ± 1-fold and 10.6 ± 0.4-fold increase in expression in H1299 and U2OS p53 Y220C–expressing cells, respectively (Fig. 4C and D; Supplementary Fig. S7A). Moreover, KG13 was found to prolong p53 Y220C occupancy on *CDKN1A* and *MDM2* promoters in H1299 and U2OS cells (Supplementary Fig. S7B and S7C). To compare KG13-mediated reactivation of p53 Y220C to p53 WT activity, we treated the U2OS cell panel with the MDM2 inhibitor Nutlin-3a and observed an 8.5 ± 0.2-fold increase in *CDKN1A* in the parental cells (Fig. 4D). The p53 Y220C–expressing U2OS cells also responded Nutlin-3a; however, the effect was suppressed due to the dominant-negative effect on p53 WT (Fig. 4D).

To test the therapeutic potential of KG13, we treated the patient-derived cells NUGC-4 (p53^+/+^), NUGC-3 (p53^Y220C/+^), and BxPC-3 (p53^Y220C/−^) with KG13, and monitored target gene expression, growth inhibition, and caspase activity. We first performed a time-course Western blot after treating the cells with DMSO, 10 μmol/L Nutlin-3a, or 10 μmol/L KG13 at 28°C, and monitored p53, p21, and MDM2 protein levels upon returning to 37°C. In DMSO-treated cells, both p53 Y220C cell lines NUGC-3 and BxPC-3 show a potent induction of MDM2 and p21 at 28°C that dissipates over the 8-hour shift to 37°C (Fig. 4E). p53 WT also shows greater activity in NUGC-4 cells at 28°C; however, the induction of MDM2 and p21 is less pronounced compared with the temperature-sensitive mutants, consistent with what has been observed previously (21). In the Nutlin-3a–treated cell panel, only p53 WT in NUGC-4 shows potent induction of MDM2 and p21 starting 4 hours after shift to 37°C (Fig. 4E). Notably, Nutlin-3a does not stimulate p53 Y220C activity even when the protein is functional at 28°C, suggesting an alternative degradation pathway for the temperature-sensitive mutant. In the KG13-treated panel, we observed elevated MDM2 and p21 protein levels in NUGC-3 and BxPC-3 compared with DMSO or Nutlin-3a–treated cells, whereas no effect was observed in the p53 WT NUGC-4 cells. In the patient-derived p53 Y220C cell panel, *BBC3* was the most upregulated gene in response to 25 μmol/L KG13, with a 5.1 ± 0.2-fold and 5.3 ± 0.8-fold increase in NUGC-3 and BxPC-3 cells, respectively (Supplementary Fig. S7D). Finally, we monitored growth inhibition and caspase-3/7 activity following KG13 treatment on the patient-derived cell lines and found an IC_50_ (compound concentration at 50% viability inhibition) of 14.7 ± 0.6 μmol/L, 7.1 ± 0.2 μmol/L, and 11.8 ± 0.6 μmol/L for NUGC-4, NUGC-3, and BxPC-3, respectively, with an increase in caspase-3/7 activity observed only in p53 Y220C cells (Fig. 4F; Supplementary Fig. S7E and S7F). In summary, KG13-treated cells displayed p53 Y220C–dependent activation of p53 target genes with growth inhibition and increased caspase activity.

## DISCUSSION

In this study, we have identified the first Y220C-specific covalent compounds that enhance mutant p53 thermal stability to WT levels. Our structural analysis revealed two conformational states of the covalently modified p53 Y220C, with the KG6-Y220C L6 state representing a novel druggable pocket. Previous Markov state models and nuclear magnetic resonance studies have proposed dynamic conformations of L6, suggesting this pocket also exists in an unliganded state (22). Although the p53 D228 backbone carbonyl represents an important docking site for the PhiKan reversible compounds (8, 9), we found that the covalent molecules presented here rely on a direct interaction with the D228 carboxylate for thermal stability and labeling efficiency (Fig. 2D and C).

Similar to the development of KRAS G12C inhibitors, the reactivity of KG13 toward the p53 somatic mutant cysteine Y220C provides a precision medicine approach to generate p53 WT activity specifically in tumor cells harboring the p53 Y220C mutation (Fig. 4C, D, and F). This approach would circumvent the toxicity observed through pan-p53 activation with MDM2 inhibitor compounds (23). Moreover, we observed that Nutlin-3a does not stimulate p53 Y220C activity at permissive temperatures when the mutant is folded in a WT-like state and activating target gene expression (Fig. 4E; Supplementary Fig. S7). The KG13 molecular chaperone mechanism of rescuing the thermal stability of the p53 DBD fold has precedent in the clinic through voxelotor, which also covalently reacts with a tetrameric target to prevent cellular aggregation (24). Future studies will be required to determine how to achieve the maximum tumor-suppressive effect from p53 Y220C–stabilizing compounds, which could be achieved through combination with cell-cycle inhibitors, proapoptotic drugs, or therapeutic hypothermia (21).

## METHODS

### Recombinant Protein Expression and Purification

Human p53 WT, Y220C, and R273C “cys-light” DBD [residues 94–312 (C124S, M133L, C182S, C229S, N239Y, N268D, C275S, C277S) p53 WT-CL, p53 Y220C-CL, p53 R273C-CL] or “non-cysteine light” [residues 94–312 (M133L, V203A, N239Y, N268D) p53 WT, p53 Y220C] were expressed as 6X HIS fusion proteins in *Escherichia coli* BL21(DE3). Bacterial cultures were grown to 0.4 OD_600_ and induced with 1 mmol/L IPTG (GoldBio) and 200 μmol/L ZnSO_4_ at 18°C for 16 hours. Cells were lysed by sonication in lysis buffer containing 50 mmol/L HEPES, 500 mmol/L NaCl, 5% glycerol, and 20 mmol/L imidazole (pH 7), clarified by centrifugation, and loaded onto Ni Sepharose High Performance (GE Healthcare) affinity resin. Following 10 CV of washing with lysis buffer, proteins were eluted from the resin in HIS elution buffer containing 50 mmol/L HEPES and 400 mmol/L imidazole (pH 7) and diluted in buffer HA containing 50 mmol/L HEPES, 20 mmol/L NaCl, and 15% glycerol (pH 7), such that the solution <100 mmol/L NaCl. The protein was then loaded onto HiTrap Heparin HP (Cytiva) affinity chromatography and eluted using buffer HA and buffer HB containing 50 mmol/L HEPES, 1 M NaCl, and 15% glycerol (pH 7), with a gradient 0% to 100% Buffer HB over 100 mL. The Heparin elution fraction was then subjected to HIS-TEV protease cleavage overnight in 50 mmol/L HEPES, 200 mmol/L NaCl, and 5 mmol/L DTT (pH 7). The protein was then passed over HiTrap Heparin HP (Cytiva) affinity resin again to remove free 6XHIS and HIS-TEV protease and was flash-frozen in liquid nitrogen.

### Crystallization, Data Collection, Structure Determination, and Model Refinement

p53 Y220C-CL was prepared for crystallization by treatment with an excess covalent compound in a buffer containing 50 mmol/L HEPES and 150 mmol/L NaCl (pH 7) for 24 hours at 4°C. The p53 Y220C-CL–small-molecule complex was then eluted from a Superdex 75 column (GE Healthcare) in a buffer containing 10 mmol/L Tris and 100 mmol/L NaCl (pH 8). The p53 Y220C-CL–small-molecule complex was crystallized from a 10 mg/mL solution by hanging drop vapor diffusion method at 22°C. Pyramidal crystals for KG3 and KG6 or rod-shaped crystals for KG10 and KG13 formed after 2 to 6 days in 100 mmol/L HEPES and 2.2M MgSO_4_ (pH 7). Crystals were cryo-protected in a reservoir solution supplemented with 25% ethylene glycol and cryo-cooled in liquid nitrogen.

Data were collected at the Advanced Light Source, Lawrence Berkeley National Laboratory, at Beamline 8.2.1. Diffraction spots were integrated using MOSFLM, and data were merged and scaled using Scala in the CCP4 software package. The model was built with Coot, and the model was refined with Phenix (25). Phenix Xtriage indicated a perfect merohedral twin for the KG3 dataset, and the -h,-k,l twin law was applied to refinement.

### Cell Lines

We used the following cell lines: H1299 (ATCC, CRL-5803), HUH-7 [University of California, San Francisco (UCSF), Jura Lab], U2OS (UCSF, cell culture facility), NUGC-3 (JCRB, JCRB0822), NUGC-4 (JCRB, JCRB0834), BxPC-3 (ATCC, CRL-1687), and HepG2 (UCSF, cell culture facility). ATCC and JCRB cell lines were tested for *Mycoplasma* prior to arrival. All UCSF cell culture facility lines were tested for endotoxin, and short tandem repeat was validated by the facility. HUH-7 cells were not tested.

### Western Blots, Immunoprecipitation, and Antibodies

Whole-cell extracts were prepared by lysing cells with lysis buffer containing 50 mmol/L HEPES, 150 mmol/L NaCl, 1 mmol/L DTT, and 0.1% NP-40 (pH 7) in the presence of 1× cOmplete EDTA-free protease inhibitor cocktail (Roche). Whole-cell extracts were combined with 2× SDS-loading buffer for Western blot analysis.

Western blots were performed with 5 μg total protein lysate and were resolved by SDS-PAGE on 4% to 12% BT gels (Invitrogen) at 150 V for 1 hour in MES buffer and transferred to nitrocellulose membranes, which were then incubated with primary antibodies at 4°C overnight, followed by incubation with LI-COR IRDye anti-mouse and anti-rabbit secondary antibodies at room temperature for 1 hour. Bands were imaged on a LI-COR Odyssey scanner.

For immunoprecipitation, cell extracts from H1299 cells were prepared by sonicating them in an immunoprecipitation (IP) lysis buffer containing 50 mmol/L Tris, 150 mmol/L NaCl, 1 mmol/L DTT, 10% glycerol, and 0.1% Tween-20 (pH 8) in the presence of 1× Sigma protease inhibitor cocktail. Total protein (400 μg) from cleared lysate was then incubated with 2 μg Pab1620 (MABE339, Millipore) for 4 hours at 4°C with 20 μL protein A/G beads in 500 μL IP lysis buffer. The mixture was then washed 3× with IP lysis buffer and eluted with 2× SDS-loading buffer for Western blot analysis.

For compound treatment (Fig. 4E), the same protocol was followed as RT-qPCR.

Antibodies used for Western blot detection were as follows: p53 (DO-1, Santa Cruz Biotechnology), p53 (7F5, Cell Signaling Technology), MDM2 (D1V2Z, Cell Signaling Technology), β-actin (8H10D10, Cell Signaling Technology), and p21 (SX118, Santa Cruz Biotechnology).

### LC-MS Analysis of p53 Y220C Covalent Labeling

Recombinant p53 (1 μmol/L) in a buffer containing 50 mmol/L HEPES and 150 mmol/L NaCl (pH 7) was treated with covalent compounds (10 μmol/L) for 24 hours at 4°C. The reactions were quenched by addition of 10 mmol/L DTT. The extent of covalent labeling was assessed by LC-MS (Waters Xevo G2-XS QTof, ACQUITY UPLC Protein BEH C4 Column, 300 Å, 1.7 μm, 2.1 mm × 50 mm). Deconvolution of multiply charged ions was performed using Waters MassLynx software (version 4.1). Reactions were performed in duplicate.

### Differential Scanning Fluorimetry

Recombinant p53 Y220C-CL was treated with an excess covalent compound in a buffer containing 50 mmol/L HEPES and 150 mmol/L NaCl (pH 7) for 24 hours at 4°C. The p53 Y220-CL–small-molecule complex was then eluted from a Superdex 75 column (GE Healthcare) in a buffer containing 50 mmol/L HEPES and 150 mmol/L NaCl (pH 7), and the peak fraction was isolated. The p53 Y220C-CL–small-molecule complex (5 μmol/L), 2× SYPRO Orange (Invitrogen), 50 mmol/L HEPES, and 150 mmol/L NaCl (pH 7) were mixed in a 96-well, white PCR plate (USA Scientific) at a volume of 25 μL per well. The plate was sealed with optically clear PCR sealing film (USA Scientific). The thermal shift assay was performed on a Bio-Rad CFX96 qPCR system. The temperature was increased from 15°C to 70°C at a rate of 0.5°C/second. After each temperature step, fluorescence was monitored with an excitation of 492 nm and an emission of 610 nm. Each sample was run in triplicate. “Cys-light” WT was analyzed under the same conditions without compound. Tm was calculated using the Boltzmann function (GraphPad Prism 8).

### RT-qPCR

Cells (0.3 × 10^6^) were plated in 6-well plates and allowed to recover for 24 hours at 37°C. Cells were transferred to 28°C for 16 hours before being treated with KG13 for 1 hour. The compound was washed out with DMEM, and the cells recovered at 37°C for 8 hours. Nutlin-3a– and DMSO-treated samples were also incubated at 28°C for 1 hour, and then left in the media during the 8-hour recovery at 37°C. RNA was isolated from cells using the RNeasy Plus Mini Kit (Qiagen, #74004) and SuperScript III First-Strand Synthesis SuperMix for qRT-PCR (Life Technologies, #11752050) was used for reverse transcription reaction. qPCR reactions were then performed using the Maxima SYBR Green qPCR Master Mix (Life Technologies, #K0221) on a Bio-Rad CFX96 qPCR system. *GAPDH* served as the reference gene. All data were evaluated using the ΔΔCt method. Primer sequences: *GAPDH* forward: 5′-GACCCCTTCATTGACCTCAAC-3′, *GAPDH* reverse: 5′-CACGACGTACTCAGCGCC-3′; *CDKN1A* forward: 5′- GGAAGACCATGTGGACCTGT-3′, *CDKN1A* reverse: 5′-GGATTAGGGCTTCCTCTTGG-3′; *MDM2* forward: 5′-GTGAATCTACAGGGACGCCA-3′, *MDM2* reverse: 5′- CTGATCCAACCAATCACCTGAA-3′; and *BBC3* forward: 5′-CCTGGAGGGTCCTGTACAATCT-3′, *BBC3* reverse: 5′-TCTGTGGCCCCTGGGTAAG-3′. Samples were run in technical triplicate and biological duplicate.

### CETSA

To generate p53 Y220C–expressing cells, H1299 cells (p53^−/−^) were transfected with Y220C-modified p53 plasmid (Addgene, #49242) using FuGENE 6 (Promega, #E2691). Stable cells were selected with G418 for 14 days. Cells (3 × 10^6^) were plated in 10-cm dishes and were allowed to recover overnight at 37°C. The cells were then transferred to a 28°C incubator for 24 hours and treated with DMSO or KG13 for 1 hour. Cells were harvested by scraping in PBS and pelleted at 3,500 rpm for 5 minutes. Cells were washed in PBS and spun down again for 5 minutes. Cells were resuspended in 450 μL PBS with 1× cOmplete EDTA-free protease inhibitor cocktail (Sigma, #11836170001) and split into 50 μL aliquots in an 8-tube strip (USA Scientific, #1402-4700). The cell suspension was heated with a gradient from 30°C to 50°C in a thermocycler for 3 minutes and then cooled to 25°C for 3 minutes. The heat-treated samples were flash-frozen in liquid nitrogen, thawed at 25°C, and briefly vortexed. The freeze–thaw cycle was repeated three times in total. Samples were cleared of aggregate by centrifugation at 20,000 × *g* and 4°C for 20 minutes. Cleared supernatant (30 μL) was mixed with 5× SDS-loading buffer for Western blot analysis. For WT p53, the same protocol was followed except H1299 cells were transiently transfected. The intensity ratio from anti-p53 rabbit (Cell Signaling Technology, #7F5) to anti-actin mouse (Cell Signaling Technology, #8H10D10) was calculated and normalized to the starting point at 30°C. Each sample was run in biological duplicate, and Tagg was calculated using the Boltzmann function (GraphPad Prism 8).

### EMSA

The p53-binding site of the *CDKN1A* promoter was assembled by heating an equimolar mixture of oligonucleotides, forward 5′-AAAGGAACATGTCCCAACATGTTGAGAA-3′ and reverse 5′-TTCTCAACATGTTGGGACATGTTCCTTT-3′, to 95°C for 5 minutes and cooling to 25°C over 1 hour. *CDKN1A* (2 μmol/L) was incubated with a p53 DBD gradient ranging from 0 to 20 μmol/L at 25°C for 30 minutes in 0.5× TBE buffer. Following incubation, samples were mixed 1:1 with 2× Native loading buffer [62.5 mmol/L Tris (pH 7), 40% glycerol, 0.01% bromophenol blue], loaded onto a DNA retardation gel (Thermo Fisher, #EC63655BOX) and run at 75V for 1 hour at 25°C. The gel was stained in 1× SYBR Safe DNA Gel Stain (Thermo Fisher, #S33102) with 0.5× TBE buffer for 10 minutes before being analyzed by a blue light transilluminator. The top intensities of the gel shift were calculated using ImageJ, and the Kd values were calculated using a specific binding function (GraphPad Prism 8).

### Cell Viability and Caspase-3/7 Assay

Cells were seeded into 96-well plates (Greiner Bio-One, #655083) at 3 × 10^3^ cells per well and were allowed to recover at 37°C overnight. Cells were then transferred to 28°C for 16 hours before being treated with the indicated compound for 1 hour. KG13 was then washed out with DMEM, and the cells were transferred to 37°C for 48 hours. Cells treated with Nutlin-3a were transferred to 37°C for 48 hours without the washout. CellTiter-Glo (Promega, #G7572) luminescence-based assay was diluted 1:5 in PBS and was mixed directly in the cell wells as a 1:1 mixture. The reaction mixture was incubated for 30 minutes while shaking at 25°C before being analyzed on a Tecan Spark plate reader. For caspase-3/7 activity, caspase-Glo 3/7 (Promega, #G8091) was added to the cells at a 1:1 mixture 24 hours following compound treatment. The mixture was incubated at 25°C for 10 minutes while shaking before being analyzed on a Tecan Spark plate reader.

### Chromatin Immunoprecipitation Assay

Cells were seeded into 150-mm dishes at 70% confluency and were allowed to recover at 37°C overnight. Cells were then transferred to 28°C for 16 hours before being treated with the indicated compound for 1 hour. KG13 was then washed out with DMEM, and the cells were transferred to 37°C for 1 or 2 hours. Cells treated with DMSO or Nutlin-3a were transferred to 37°C for 1 or 2 hours without the washout. Cells were harvested and cross-linked in 1% formaldehyde in PBS for 10 minutes at room temperature. The Zymo-Spin ChIP Kit (Zymo Research, #D5210) was used for DNA IP and purification, except agarose beads (Santa Cruz Biotechnology, #sc-2003) were used instead of magnetic beads. For sonication, a Bioruptor Pico sonicator (Diagenode, #B01060010) was used with mode set at 20 cycles, 30 seconds on/off. For p53 IP, DO-1 (Santa Cruz Biotechnology, #sc-126) and normal mouse IgG (Santa Cruz Biotechnology, #2025) were used with enrichment calculated as 2‸-(DO-1 Cq – IgG Cq). qPCR reactions were performed using the Maxima SYBR Green qPCR Master Mix (Life Technologies) on a Bio-Rad CFX96 qPCR system. Chromatin immunoprecipitation (ChIP) primer sequences were as follows: *CDKN1A* forward: 5′-AGCAGGCTGTGGCTCTGATT-3′, *CDKN1A* reverse: 5′-CAAAATAGCCACCAGCCTCTTCT-3′; *MDM2* forward: 5′-CGTTCCGAAACTGCAGTAAA-3′, *MDM2* reverse: 5′-CAGCTGGAGACAAGTCAGGA-3′. Each sample was run in biological and technical duplicates.

### Compounds

We used PhiKan083 (Sigma-Aldrich, #SML1770), PK11000 (Enamine, #EN300–27043), and Nutlin-3a (Apexbio, #A3671).

#### 1-Carbazol-9-Ylprop-2-En-1-One (KG1).

An oven-dried reaction vessel was placed under argon and charged with 9H-carbazole (100 mg, 0. 0.598 mmol/L) and sodium hydride (21.53 mg, 0.897 mmol/L). THF (2 mL) was added, and the reaction was stirred at 4°C for 10 minutes. Prop-2-enoyl prop-2-enoate (113.1 mg, 0.897 mmol/L) was added, and the reaction was warmed from 4°C to room temperature over 30 minutes. The reaction mixture was quenched with saturated sodium bicarbonate solution and extracted in dichloromethane. The mixture was washed in a brine solution, dried with sodium sulfate, and concentrated *in vacuo*. The crude was purified by flash chromatography over silica gel eluting with a gradient from 0% to 15% ethyl acetate-hexanes to afford KG1 (21 mg, 0.095 mmol/L, 15.87% yield) as a white semisolid. ^1^H NMR (400 MHz, DMSO-*d*_6_) δ 8.22 (ddd, *J* = 7.6, 1.4, 0.7 Hz, 2H), 8.12 (dt, *J* = 8.4, 0.9 Hz, 2H), 7.54 (ddd, *J* = 8.5, 7.3, 1.4 Hz, 2H), 7.45 (td, *J* = 7.5, 1.0 Hz, 2H), 7.27 (dd, *J* = 16.9, 10.6 Hz, 1H), 6.55 (dd, *J* = 16.9, 1.5 Hz, 1H), 6.18 (dd, *J* = 10.5, 1.5 Hz, 1H). HRMS (ESI-TOF): m/z: calculated for C_16_H_11_NO_2_ [M + H]+ 249.3, found 250.0866.

#### 1-Carbazol-9-Yl-2-Chloro-Ethanone (KG2).

An oven-dried reaction vessel was placed under argon and charged with 9H-carbazole (100 mg, 0. 0.598 mmol/L) and sodium hydride (21.53 mg, 0.897 mmol/L). THF (2 mL) was added, and the reaction was stirred at 4°C for 10 minutes. Chloroacetyl chloride (101.3, 0.897 mmol/L) was added, and the reaction was warmed from 4°C to room temperature over 30 minutes. The reaction mixture was quenched with saturated sodium bicarbonate solution and extracted in dichloromethane. The mixture was washed in a brine solution, dried with sodium sulfate, and concentrated *in vacuo*. The crude was purified by flash chromatography over silica gel eluting with a gradient from 0% to 15% ethyl acetate-hexanes to afford KG2 (9.9 mg, 0.041 mmol/L, 6.793% yield) as a clear semisolid. ^1^H NMR (400 MHz, DMSO-*d*_6_) δ 8.50 – 8.12 (m, 4H), 7.75 – 7.38 (m, 4H), 5.35 (s, 2H). HRMS (ESI-TOF): m/z: calculated for C_15_H_11_NO [M + H]+ 221.3, found 222.0939.

#### 9-Prop-2-Enoylcarbazole-3-Carbaldehyde (KG3).

An oven-dried reaction vessel was placed under argon and charged with 9H-carbazole-3-carbaldehyde (100 mg, 0.512 mmol/L). Dichloromethane (2 mL), triethylamine (214.19 μL, 1.537 mmol/L), and prop-2-enoyl prop-2-enoate (70.47 μL, 0.615 mmol/L) were added, and the reaction was stirred at room temperature for 1 hour under argon. The reaction mixture was quenched with saturated sodium bicarbonate solution and extracted in dichloromethane. The mixture was washed in the brine solution, dried with sodium sulfate, and concentrated *in vacuo*. The crude was purified by flash chromatography over silica gel eluting with a gradient from 0% to 50% ethyl acetate-hexanes to afford KG3 (25 mg, 0.1 mmol/L, 19.58% yield) as a yellow semisolid. ^1^H NMR (400 MHz, DMSO-*d*_6_) δ 10.15 (s, 1H), 8.81 (dd, *J* = 1.7, 0.6 Hz, 1H), 8.42 – 8.26 (m, 2H), 8.15 – 8.05 (m, 2H), 7.66 – 7.47 (m, 2H), 7.27 (dd, *J* = 16.9, 10.6 Hz, 1H), 6.60 (dd, *J* = 16.9, 1.5 Hz, 1H), 6.24 (dd, *J* = 10.6, 1.4 Hz, 1H). HRMS (ESI-TOF): m/z: calculated for C_14_H_10_ClNO [M + H]+ 243.7, found 244.0532.

#### 9-Prop-2-Enoylcarbazole-3-Carbaldehyde (KG4).

An oven-dried reaction vessel was placed under argon and charged with 9H-carbazole-3-carbaldehyde (100 mg, 0.512 mmol/L) and N-Methylpropargyl amine (106.2 mg, 1.537 mmol/L). Methanol (1.8 mL) and glacial acetic acid (0.2 mL) were added, and the reaction was stirred at room temperature for 30 minutes. Sodium cyanoborohydride (38.63 mg, 0.615 mmol/L) was added, and the reaction proceeded overnight. The reaction mixture was quenched with saturated sodium bicarbonate solution and extracted in ethyl acetate. The mixture was washed in the brine solution, dried with sodium sulfate, and concentrated *in vacuo*. The crude was purified by flash chromatography over silica gel eluting with a gradient from 0% to 100% ethyl acetate-hexanes to afford N-(9H-carbazol-3-ylmethyl)-N-methyl-prop-2-yn-1-amine (108 mg, 0.435 mmol/L, 84.91% yield) as a white/yellow semisolid and was used in the next reaction step. An oven-dried reaction vessel was placed under argon and charged with N-(9H-carbazol-3-ylmethyl)-N-methyl-prop-2-yn-1-amine (108 mg, 0.435 mmol/L) and sodium hydride (20.88 mg, 0.870 mmol/L). THF (2 mL) was added, and the reaction was stirred at 4°C for 10 minutes. Prop-2-enoyl prop-2-enoate (82.27 mg, 0.652 mmol/L) was added, and the reaction was warmed from 4°C to room temperature over 30 minutes. The reaction mixture was quenched with saturated sodium bicarbonate solution and extracted in dichloromethane. The mixture was washed in a brine solution, dried with sodium sulfate, and concentrated *in vacuo*. The crude was purified by flash chromatography over silica gel eluting with a gradient from 0% to 80% ethyl acetate-hexanes to afford KG4 (3.9 mg, 0.013 mmol/L, 2.966% yield) as a clear semisolid. ^1^H NMR (400 MHz, DMSO-*d*_6_) δ 8.53 – 8.00 (m, 3H), 7.71 – 7.09 (m, 3H), 6.54 (dd, *J* = 16.9, 1.6 Hz, 1H), 6.18 (dd, *J* = 10.5, 1.6 Hz, 1H), 3.69 (s, 2H), 3.23 (t, *J* = 2.4 Hz, 1H), 2.26 (s, 2H), 1.24 (d, *J* = 3.1 Hz, 3H), 0.86 (t, *J* = 6.5 Hz, 1H). HRMS (ESI-TOF): m/z: calculated for C_20_H_18_N_2_O [M + H]+ 302.4, found 235.0956 (loss of NCH_3_CH_2_CCH).

#### 9-Prop-2-Enoyl-N-prop-2-Ynyl-Carbazole-3-Carboxamide (KG5).

An oven-dried reaction vessel was placed under argon and charged with 9H-carbazole-3-carboxylic acid (200 mg, 0.947 mmol/L) and HATU (0.947 mg, 1.42 mmol/L). DMF (2 mL) and N,N-diisopropylpropan-2-amine (407 mg, 2.841 mmol/L) were added, and the reaction was stirred at room temperature for 10 minutes under argon. Prop-2-yn-1-amine (104.3 mg, 1.894 mmol/L) was added, and the reaction proceeded overnight. The product was extracted in ethyl acetate, washed in 5% citric acid solution, brine solution, sodium bicarbonate solution, brine solution, dried with sodium sulfate, and concentrated *in vacuo*. The crude was purified by flash chromatography over silica gel eluting with a gradient from 0% to 100% ethyl acetate-hexanes to afford N-prop-2-ynyl-9H-carbazole-3-carboxamide (225 mg, 0.906 mmol/L, 95.71% yield) as a white/yellow semisolid and was used in the next reaction step. An oven-dried reaction vessel was placed under argon and charged with N-prop-2-ynyl-9H-carbazole-3-carboxamide (100 mg, 0.403 mmol/L). Dichloromethane (2 mL), triethylamine (122.3 mg, 1.208 mmol/L), and prop-2-enoyl prop-2-enoate (60.95 mg, 0.483 mmol/L) were added, and the reaction was stirred at room temperature for 1 hour under argon. The reaction mixture was quenched with saturated sodium bicarbonate solution and extracted in dichloromethane. The mixture was washed in a brine solution, dried with sodium sulfate, and concentrated *in vacuo*. The crude was purified by flash chromatography over silica gel eluting with a gradient from 0% to 50% ethyl acetate-hexanes to afford KG5 (72 mg, 0.238 mmol/L, 59.13% yield) as a yellow semisolid. ^1^H NMR (400 MHz, DMSO-*d*_6_) δ 9.07 (t, *J* = 5.5 Hz, 1H), 8.74 (d, *J* = 1.7 Hz, 1H), 8.28 – 8.00 (m, 4H), 7.63 – 7.43 (m, 2H), 7.27 (dd, *J* = 16.9, 10.5 Hz, 1H), 6.58 (dd, *J* = 17.0, 1.5 Hz, 1H), 6.22 (dd, *J* = 10.5, 1.5 Hz, 1H), 4.14 (dd, *J* = 5.6, 2.5 Hz, 2H), 3.17 (t, *J* = 2.5 Hz, 1H). HRMS (ESI-TOF): m/z: calculated for C_19_H_14_N_2_O_2_ [M + H]+ 302.3, found 303.116.

#### 1-(2-Methylprop-2-Enoyl)indole-3-Carbaldehyde (KG6).

An oven-dried reaction vessel was placed under argon and charged with 1H-indole-3-carbaldehyde (50 mg, 0.344 mmol/L). Dichloromethane (2 mL), triethylamine (104.6 mg, 1.033 mmol/L), and methacrylic anhydride (63.72 mg, 0.413 mmol/L) were added, and the reaction was stirred at room temperature 1 hour under argon. The reaction mixture was quenched with saturated sodium bicarbonate solution and extracted in dichloromethane. The mixture was washed in brine solution, dried with sodium sulfate, and concentrated *in vacuo*. The crude was purified by flash chromatography over silica gel eluting with a gradient from 0% to 50% ethyl acetate-hexanes to afford KG6 (55.4 mg, 0.260 mmol/L, 75.44% yield) as a white semisolid. ^1^H NMR (400 MHz, Chloroform-*d*) δ 10.18 (s, 1H), 8.48 (dt, *J* = 8.4, 1.0 Hz, 1H), 8.35 – 8.29 (m, 1H), 8.20 (s, 1H), 7.55 – 7.43 (m, 2H), 7.04 (dd, *J* = 16.8, 10.4 Hz, 1H), 6.80 (dd, *J* = 16.7, 1.2 Hz, 1H), 6.22 (dd, *J* = 10.5, 1.2 Hz, 1H), 1.28 (s, 1H), 0.88 (d, *J* = 17.5 Hz, 1H). HRMS (ESI-TOF): m/z: calculated for C_13_H_11_NO_2_ [M + H]+ 213.2, found 214.0885.

#### 4-Methyl-1-(2-Methylprop-2-Enoyl)Indole-3-Carbaldehyde (KG7).

An oven-dried reaction vessel was placed under argon and charged with 4-methyl-1H-indole-3-carbaldehyde (100 mg, 0.628 mmol/L). Dichloromethane (2 mL), triethylamine (190.7 mg, 1.885 mmol/L), and methacrylic anhydride (116.2 mg, 0.754 mmol/L) were added, and the reaction was stirred at room temperature 1 hour under argon. The reaction mixture was quenched with saturated sodium bicarbonate solution and extracted in dichloromethane. The mixture was washed in brine solution, dried with sodium sulfate, and concentrated *in vacuo*. The crude was purified by flash chromatography over silica gel eluting with a gradient from 0% to 50% ethyl acetate-hexanes to afford KG7 (73.4 mg, 0.323 mmol/L, 51.41% yield) as a white semisolid. ^1^H NMR (400 MHz, DMSO-*d*_6_) δ 10.06 (s, 1H), 8.58 (s, 1H), 8.13 (dt, *J* = 8.3, 0.9 Hz, 1H), 7.36 (dd, *J* = 8.3, 7.4 Hz, 1H), 7.22 (dt, *J* = 7.4, 1.0 Hz, 1H), 5.97 (q, *J* = 1.6 Hz, 1H), 5.68 (d, *J* = 1.2 Hz, 1H), 2.78 (s, 3H), 2.14 (dd, *J* = 1.6, 1.0 Hz, 3H). HRMS (ESI-TOF): m/z: calculated for C_14_H_13_NO_2_ [M + H]+ 227.3, found 228.1046.

#### 1-(2-Methylprop-2-Enoyl)-4-Pyrimidin-5-Yl-indole-3-Carbaldehyde (KG8).

An oven-dried reaction vessel was placed under argon and charged with 4-bromo-1H-indole-3-carbaldehyde (100 mg, 0.446 mmol/L), cesium carbonate (290.8 mg, 0.893 mmol/L), 1,1′-Bis(di-tert-butylphosphino)ferrocene-palladium dichloride (14.63 mg, 0.050 mmol/L), and pyrimidin-5-ylboronic acid (82.96 mg, 0.669 mmol/L). Water (1 mL) and acetonitrile (1 mL) were degassed with argon and were added to the reaction vessel. The reaction was stirred at 80°C for 24 hours, cooled to room temperature, and extracted in ethyl acetate, washed in a brine solution, dried with sodium sulfate, and concentrated *in vacuo*. The crude was purified by flash chromatography over silica gel eluting with a gradient from 0% to 100% ethyl acetate-hexanes to afford 4-pyrimidin-5-yl-1H-indole-3-carbaldehyde (20 mg, 0.090 mmol/L, 20.07% yield) as a brown/orange solid and was used in the next reaction step. An oven-dried reaction vessel was placed under argon and charged with 4-pyrimidin-5-yl-1H-indole-3-carbaldehyde (20 mg, 0.090 mmol/L). Dichloromethane (2 mL), triethylamine (27.2 mg, 0.269 mmol/L), and methacrylic anhydride (16.57 mg, 0.108 mmol/L) were added, and the reaction was stirred at room temperature for 1 hour under argon. The reaction mixture was quenched with saturated sodium bicarbonate solution and extracted in dichloromethane. The mixture was washed in a brine solution, dried with sodium sulfate, and concentrated *in vacuo*. The crude was purified by flash chromatography over silica gel eluting with a gradient from 0% to 50% ethyl acetate-hexanes to afford KG8 (12 mg, 0.8024 mmol, 78% yield) as a yellow semisolid. ^1^H NMR (400 MHz, DMSO-*d*_6_) δ 9.77 (s, 1H), 9.21 (s, 1H), 8.78 (d, *J* = 9.0 Hz, 3H), 8.43 (dd, *J* = 8.4, 1.0 Hz, 1H), 7.69–7.60 (m, 1H), 7.46 (dd, *J* = 7.5, 1.0 Hz, 1H), 6.03 (q, *J* = 1.6 Hz, 1H), 2.17 (d, *J* = 1.3 Hz, 3H), 1.84 (t, *J* = 1.3 Hz, 1H). HRMS (ESI-TOF): m/z: calculated for C_17_H_13_N_3_O_2_ [M + H]+ 291.3, found 292.1141.

#### 4-[2-(4-Methylpiperazin-1-yl)Pyrimidin-5-yl]-1-(2-Methylprop-2-enoyl)Indole-3-Carbaldehyde (KG9).

An oven-dried reaction vessel was placed under argon and charged with 4-bromo-1H-indole-3-carbaldehyde (400 mg, 1.785 mmol/L), cesium carbonate (1,163 mg, 3.571 mmol/L), 1,1′-Bis(di-tert-butylphosphino)ferrocene-palladium dichloride (58.54 mg, 0.089 mmol/L), and 2-(4-methylpiperazin-1-yl)-5-(4,4,5,5-tetramethyl-1,3,2-dioxaborolan-2-yl)pyrimidine (814.6 mg, 2.678 mmol/L). Water (2 mL) and acetonitrile (2 mL) were degassed with argon and were added to the reaction vessel. The reaction was stirred at 80°C for 24 hours, cooled to room temperature, and extracted in ethyl acetate, washed in a brine solution, dried with sodium sulfate, and concentrated *in vacuo*. The crude was purified by flash chromatography over silica gel eluting with a gradient from 0% to 20% methanol-dichloromethane to afford 4-[2-(4-methylpiperazin-1-yl)pyrimidin-5-yl]-1H-indole-3-carbaldehyde (443.9 mg, 1.381 mmol, 77.37% yield) as a brown/red solid and was used in the next reaction step. An oven-dried reaction vessel was placed under argon and charged with 4-[2-(4-methylpiperazin-1-yl)pyrimidin-5-yl]-1H-indole-3-carbaldehyde (100 mg, 0.311 mmol/L). Dichloromethane (2 mL), triethylamine (94.46 mg, 0.933 mmol/L), and methacrylic anhydride (57.56 mg, 0.373 mmol/L) were added, and the reaction was stirred at room temperature for 1 hour under argon. The reaction mixture was quenched with saturated sodium bicarbonate solution and extracted in dichloromethane. The mixture was washed in a brine solution, dried with sodium sulfate, and concentrated *in vacuo*. The crude was purified by flash chromatography over silica gel eluting with a gradient from 0% to 20% methanol-dichloromethane to afford KG9 (65 mg, 0.167 mmol, 53.64% yield) as a brown semisolid. ^1^H NMR (400 MHz, DMSO-*d*_6_) δ 12.44 (s, 1H), 9.70 (s, 1H), 8.36 (d, *J* = 2.0 Hz, 3H), 7.55 (d, *J* = 8.0 Hz, 1H), 7.35 (t, *J* = 7.7 Hz, 1H), 7.12 (d, *J* = 7.3 Hz, 1H), 5.97 (s, 1H), 5.60 (t, *J* = 1.9 Hz, 1H), 3.80 (t, *J* = 4.9 Hz, 4H), 2.41 (t, *J* = 5.0 Hz, 4H), 2.25 (s, 3H), 1.85 (s, 2H). HRMS (ESI-TOF): m/z: calculated for C_22_H_24_N_5_O_2_^+^ [M + H]+ 390.19, found 390.2006.

#### 4-[4-(4-Methylpiperazin-1-yl)Phenyl]-1-(2-Methylprop-2-Enoyl)Indole-3-Carbaldehyde (KG10).

An oven-dried reaction vessel was placed under argon and charged with 4-bromo-1H-indole-3-carbaldehyde (400 mg, 1.785 mmol/L), cesium carbonate (1163 mg, 3.571 mmol/L), 1,1′-Bis(di-tert-butylphosphino)ferrocene-palladium dichloride (58.54 mg, 0.089 mmol/L), and 1-methyl-4-[4-(4,4,5,5-tetramethyl-1,3,2-dioxaborolan-2-yl)phenyl]piperazine (809.3 mg, 2.678 mmol/L). Water (2 mL) and acetonitrile (2 mL) were degassed with argon and were added to the reaction vessel. The reaction was stirred at 80°C for 24 hours, cooled to room temperature, and extracted in ethyl acetate, washed in a brine solution, dried with sodium sulfate, and concentrated *in vacuo*. The crude was purified by flash chromatography over silica gel eluting with a gradient from 0% to 20% methanol-dichloromethane to afford 4-[4-(4-methylpiperazin-1-yl)phenyl]-1H-indole-3-carbaldehyde (266 mg, 0.833 mmol/L, 46.65% yield) as a brown/red solid and was used in the next reaction step. An oven-dried reaction vessel was placed under argon and charged with 4-[4-(4-methylpiperazin-1-yl)phenyl]-1H-indole-3-carbaldehyde (100 mg, 0.313 mmol/L). Dichloromethane (2 mL), triethylamine (95.04 mg, 0.939 mmol/L), and methacrylic anhydride (57.92 mg, 0.376 mmol/L) were added, and the reaction was stirred at room temperature for 1 hour under argon. The reaction mixture was quenched with saturated sodium bicarbonate solution and extracted in dichloromethane. The mixture was washed in a brine solution, dried with sodium sulfate, and concentrated *in vacuo*. The crude was purified by flash chromatography over silica gel eluting with a gradient from 0% to 20% methanol-dichloromethane to afford KG10 (76 mg, 0.196 mmol, 62.65% yield) as a brown semisolid. ^1^H NMR (400 MHz, DMSO-*d*_6_) δ 8.34 – 8.22 (m, 1H), 7.58 – 7.27 (m, 5H), 7.12 – 6.97 (m, 3H), 6.04 – 5.93 (m, 1H), 5.60 (q, *J* = 1.7 Hz, 1H), 3.22 (q, *J* = 5.2 Hz, 6H), 2.25 (s, 3H), 2.12 (s, 1H), 1.85 (t, *J* = 1.2 Hz, 2H). HRMS (ESI-TOF): m/z: calculated for C_24_H_27_N_3_O_2_^2+^ [M + H]+ 389.21, found 389.2105

#### 2-Methyl-1-[3-Methyl-4-[4-(4-Methylpiperazin-1-yl)Phenyl]Pyrrolo[2,3-c]Pyridin-1-yl]Prop-2-en-1-one (KG11).

An oven-dried reaction vessel was placed under argon and charged with 4-bromo-3-methyl-1H-pyrrolo[2,3-c]pyridine (200 mg, 0.948 mmol/L), cesium carbonate (617.5 mg, 1.895 mmol/L), 1,1′-Bis(di-tert-butylphosphino)ferrocene-palladium dichloride (31.07 mg, 0.047 mmol/L), and 1-methyl-4-[4-(4,4,5,5-tetramethyl-1,3,2-dioxaborolan-2-yl)phenyl]piperazine (429.6 mg, 1.421 mmol/L). Water (2 mL) and acetonitrile (2 mL) were degassed with argon and were added to the reaction vessel. The reaction was stirred at 80°C for 24 hours, cooled to room temperature, and extracted in ethyl acetate, washed in a brine solution, dried with sodium sulfate, and concentrated *in vacuo*. The crude was purified by flash chromatography over silica gel eluting with a gradient from 0% to 20% methanol-dichloromethane to afford 3-methyl-4-[4-(4-methylpiperazin-1-yl)phenyl]-1H-pyrrolo[2,3-c]pyridine (127 mg, 0.414 mmol, 43.69% yield) as a white/green solid and was used in the next reaction step. An oven-dried reaction vessel was placed under argon and charged with 3-methyl-4-[4-(4-methylpiperazin-1-yl)phenyl]-1H-pyrrolo[2,3-c]pyridine (127 mg, 0.414 mmol/L) and sodium hydride (19.9 mg, 0.829 mmol/L). DMF (2 mL) was added, and the reaction was stirred at 4°C for 10 minutes under argon. Methacrylic anhydride (69.71 μL, 0.497 mmol/L) was added at 4°C, and the reaction mixture was warmed to room temperature over 1 hour. The reaction mixture was quenched with saturated sodium bicarbonate solution and extracted in dichloromethane, dried with sodium sulfate, and concentrated *in vacuo*. The crude was purified by HPLC eluting with a gradient from 5% to 50% water/acetonitrile with 0.1% formic acid. HPLC fractions were pooled and the pH was adjusted with saturated sodium bicarbonate solution, and the product was extracted in ethyl acetate, dried with sodium sulfate, and concentrated *in vacuo* to afford KG11 (85 mg, 0. 0.227 mmol, 54.76% yield) as a yellow solid. ^1^H NMR (400 MHz, DMSO-*d*_6_) δ 9.46 (s, 1H), 8.21 (s, 1H), 7.70 (d, *J* = 1.4 Hz, 1H), 7.32 – 7.26 (m, 2H), 7.08 – 7.01 (m, 2H), 5.84 (q, *J* = 1.6 Hz, 1H), 5.57 (d, *J* = 1.2 Hz, 1H), 3.22 (dd, *J* = 6.3, 3.8 Hz, 4H), 2.48 (d, *J* = 5.0 Hz, 5H), 2.25 (s, 3H), 2.12 (d, *J* = 1.3 Hz, 3H), 1.87 (d, *J* = 1.3 Hz, 3H). HRMS (ESI-TOF): m/z: calculated for C_23_H_27_N4O^+^ [M + H]+ 375.22, found 375.2213.

#### 2-Methyl-1-[3-Methyl-4-[4-(1-Methyl-4-Piperidyl)Phenyl]Pyrrolo[2,3-c]Pyridin-1-yl]Prop-2-en-1-one (KG12).

An oven-dried reaction vessel was placed under argon and charged with 4-bromo-3-methyl-1H-pyrrolo[2,3-c]pyridine (200 mg, 0.948 mmol/L), cesium carbonate (617.5 mg, 1.895 mmol/L), 1,1′-Bis(di-tert-butylphosphino)ferrocene-palladium dichloride (31.07 mg, 0.047 mmol/L), and 1-methyl-4-[4-(4,4,5,5-tetramethyl-1,3,2-dioxaborolan-2-yl)phenyl]piperidine (285.4 mg, 0.948 mmol/L). Water (2 mL) and acetonitrile (2 mL) were degassed with argon and were added to the reaction vessel. The reaction was stirred at 80°C for 24 hours, cooled to room temperature, and extracted in ethyl acetate, washed in brine solution, dried with sodium sulfate, and concentrated *in vacuo*. The crude was purified by flash chromatography over silica gel eluting with a gradient from 0% to 20% methanol-dichloromethane to afford 3-methyl-4-[4-(1-methyl-4-piperidyl)phenyl]-1H-pyrrolo[2,3-c]pyridine (133.1 mg, 0.436 mmol/L, 45.99% yield) as a white/brown semisolid and was used in the next reaction step. An oven-dried reaction vessel was placed under argon and charged with 3-methyl-4-[4-(1-methyl-4-piperidyl)phenyl]-1H-pyrrolo[2,3-c]pyridine (133.1 mg, 0.436 mmol/L) and sodium hydride (20.92 mg, 0.872 mmol/L). DMF (2 mL) was added and the reaction was stirred at 4°C for 10 minutes under argon. Methacrylic anhydride (73.29 μL, 0.523 mmol/L) was added at 4°C, and the reaction mixture was warmed to room temperature over 1 hour. The reaction mixture was quenched with saturated sodium bicarbonate solution and extracted in dichloromethane, dried with sodium sulfate, and concentrated *in vacuo*. The crude was purified by HPLC eluting with a gradient from 5% to 50% water/acetonitrile with 0.1% formic acid. HPLC fractions were pooled and the pH was adjusted with saturated sodium bicarbonate solution, and the product was extracted in ethyl acetate, dried with sodium sulfate, and concentrated *in vacuo* to afford KG12 (71 mg, 0. 0.190 mmol/L, 43.62% yield) as a white solid. ^1^H NMR (400 MHz, DMSO-*d*_6_) δ 9.49 (s, 1H), 8.24 (s, 1H), 7.72 (t, *J* = 1.3 Hz, 1H), 7.38 (s, 4H), 5.84 (t, *J* = 1.7 Hz, 1H), 5.57 (s, 1H), 2.90 (dt, *J* = 12.0, 3.2 Hz, 2H), 2.30 – 2.06 (m, 7H), 2.00 (td, *J* = 11.5, 2.6 Hz, 2H), 1.91 – 1.62 (m, 8H). HRMS (ESI-TOF): m/z: calculated for C_24_H_28_N_3_O^+^ [M + H]+ 374.22, found 374.2265.

#### 2-Methyl-1-[3-Methyl-4-(2-Methyl-3,4-Dihydro-1H-isoquinolin-6-yl)Pyrrolo[2,3-c]Pyridin-1-yl]Prop-2-en-1-one (KG13).

An oven-dried reaction vessel was placed under argon and charged with 4-bromo-3-methyl-1H-pyrrolo[2,3-c]pyridine (200 mg, 0.948 mmol/L), cesium carbonate (617.5 mg, 1.895 mmol/L), 1,1′-Bis(di-tert-butylphosphino)ferrocene-palladium dichloride (31.07 mg, 0.047 mmol/L), and 2-methyl-6-(4,4,5,5-tetramethyl-1,3,2-dioxaborolan-2-yl)-3,4-dihydro-1H-isoquinoline (388.3 mg, 1.421 mmol/L). Water (2 mL) and acetonitrile (2 mL) were degassed with argon and were added to the reaction vessel. The reaction was stirred at 80°C for 24 hours, cooled to room temperature, and extracted in ethyl acetate, washed in a brine solution, dried with sodium sulfate, and concentrated *in vacuo*. The crude was purified by flash chromatography over silica gel eluting with a gradient from 0% to 20% methanol-dichloromethane to afford 2-methyl-6-(3-methyl-1H-pyrrolo[2,3-c]pyridin-4-yl)-3,4-dihydro-1H-isoquinoline (162.6 mg, 0.586 mmol/L, 61.87% yield) as a brown semisolid and was used in the next reaction step. An oven-dried reaction vessel was placed under argon and charged with 2-methyl-6-(3-methyl-1H-pyrrolo[2,3-c]pyridin-4-yl)-3,4-dihydro-1H-isoquinoline (162.6 mg, 0.586 mmol/L) and sodium hydride (28.14 mg, 1.172 mmol/L). DMF (2 mL) was added, and the reaction was stirred at 4°C for 10 minutes under argon. Methacrylic anhydride (98.59 μL, 0.703 mmol/L) was added at 4°C and the reaction mixture was warmed to room temperature over 1 hour. The reaction mixture was quenched with saturated sodium bicarbonate solution and extracted in dichloromethane, dried with sodium sulfate, and concentrated *in vacuo*. The crude was purified by HPLC eluting with a gradient from 5% to 50% water/acetonitrile with 0.1% formic acid. HPLC fractions were pooled and the pH was adjusted with saturated sodium bicarbonate solution, and the product was extracted in ethyl acetate, dried with sodium sulfate, and concentrated *in vacuo* to afford KG13 (71 mg, 0. 0.190 mmol/L, 43.62% yield) as a white solid. 1H NMR (400 MHz, DMSO-*d*6) δ 9.49 (s, 1H), 8.23 (s, 1H), 7.72 (d, *J* = 1.4 Hz, 1H), 7.27 – 7.12 (m, 3H), 5.85 (t, *J* = 1.7 Hz, 1H), 5.57 (s, 1H), 3.56 (s, 2H), 2.90 (t, *J* = 5.9 Hz, 2H), 2.64 (t, *J* = 5.9 Hz, 2H), 2.38 (s, 3H), 2.12 (d, *J* = 1.3 Hz, 3H), 1.83 (d, *J* = 1.3 Hz, 3H). HRMS (ESI-TOF): m/z: calculated for C_22_H_24_N_3_O^+^ [M + H]+ 346.19, found 346.1946.

### Data Availability

The crystal structures described in this study can be accessed at https://www.rcsb.org/ under accession codes 8DC4 (KG3), 8DC6 (KG6), 8DC7 (KG10), and 8DC8 (KG13).

## Supplementary Material

Table S1 X-ray crystallography data collection and refinement statistics

Supplementary Figure 1 Supplementary Figure S1: p53 hotspot mutations (A) Crystal structure of p53 bound to DNA PDB 1TUP with structural and DNA-contact mutations highlighted. (B) Frequency of hotspot mutations out of total p53 alterations based upon data generated by the TCGA Research Network: https://www.cancer.gov/tcga. (C) The urea-denaturation free energy values from hotspot mutations and stabilizing mutations plotted from previous studies (7). (D) SDS-PAGE of recombinant protein used in study stained with Coomassie blue.

Supplementary Figure 2 Supplementary Figure S2: Differential scanning fluorimetry (A) Carbazole series plot of average values from differential scanning fluorimetry. (B) Indole series plot of average values from differential scanning fluorimetry. (C) Azaindole series plot of average values from differential scanning fluorimetry. (D) p53 Y220C-CL D228A DMSO or KG11 adduct plot of average values from differential scanning fluorimetry.

Supplementary Figure 3 Supplementary Figure S3: Example LC/MS spectra from carbazole and indole series (A) LC/MS spectra from p53 WT-CL or p53 Y220C-CL labeling with KG1. (B) LC/MS spectra from p53 WT or p53 Y220C labeling with KG5. (C) LC/MS spectra from p53 WT or p53 Y220C labeling with PK11000. (D) LC/MS spectra from p53 WT or p53 Y220C labeling with KG6. (E) LC/MS spectra from p53 WT or p53 Y220C labeling with KG13.

Supplementary Figure 4 Supplementary Figure S4: Ligand electron density maps (A) 1σ 2Fo-Fc electron density map and 1.5σ Fo-Fc simulated annealing omit density map for KG3. (B) 1σ 2Fo-Fc electron density map and 1.5σ Fo-Fc simulated annealing omit density map for KG6. (C) 1σ 2Fo-Fc electron density map and 1.5σ Fo-Fc simulated annealing omit density map for KG10. (D) 1σ 2Fo-Fc electron density map and 1.5σ Fo-Fc simulated annealing omit density map for KG13. (E) Allosteric stabilization by KG6. D228 makes intramolecular contacts with V147 amine rather through a small molecule directly.

Supplementary Figure 5 Supplementary Figure S5: CDKN1A EMSA gels and intensity plots (A) EMSA for p53 WT-CL. (B) EMSA for p53 Y220C-CL. (C) EMSA for p53 Y220C-CL complexed with KG13. (D) EMSA for p53 R273C-CL. (E) Upper band intensities plotted for each protein construct. (F) Upper band intensities plotted for p53 R273C-CL.

Supplementary Figure 6 Supplementary Figure S6: Western blots and for 28°C IP and CETSA (A) H1299 cells stably expressing p53 Y220C or p53 R273C were transferred from 37°C to 28°C and p21/MDM2 levels were monitored by western blot at the indicated time points. (B) U2OS cells stably expressing p53 Y220C or parent were transferred to a 28°C incubator and p21/MDM2 levels were observed by western blot at the indicated time points. (C) Extracts from H1299 cells expressing p53 Y220C incubated at 37°C or 28°C for 24 hr were immunoprecipitated with the WT conformation antibody Pab1620. (D) CETSA western blot for p53 Y220C DMSO. (E) CETSA western blot for p53 Y220C treated with 100 μM KG13 for 1 hr. (F) CETSA western blot for p53 WT DMSO.

Supplementary Figure 7 Supplementary Figure S7: Extended p53 Y220C KG13 cellular activity (A) Western blot for isogenic cell panel treated with 10 μM Nutlin-3a constant or 10 μM KG13 for 1 hr at 28°C, washed out, then moved to 37°C for 8hr. (B) ChIP p53 enrichment for U2OS cells expressing p53 Y220C. (C) p53 ChIP enrichment at CDKN1A and MDM2 promoters relative to IgG following KG13 treatment in H1299 cells expressing p53 Y220C. (D) RT-qPCR results for 25 μM KG13 treated NUGC-4, NUGC-3, and BxPC-3ß cells. (E) Viability assay for KG13 treatment. (F) Viability assay for Nutlin-3a treatment.
