# Supplementary material for: A small molecule reacts with the p53 somatic mutant Y220C to rescue wild-type thermal stability
Source: Cancer Discov. Author manuscript; Available in PMC 2023 Jan 14. (PMC9827106; doi:10.1158/2159-8290.CD-22-0381)
Supplement: 1 [file NIHMS1842090-supplement-1.docx]

**X-ray crystallography data collection and refinement statistics**

Values in parentheses are for highest resolution shell.

Table S1.

|  | p53 Y220C-KG3 | p53 Y220C-KG6 | p53 Y220C-KG10 | P53 Y220C-KG13 |
| --- | --- | --- | --- | --- |
| Data collection |  |  |  |  |
| Space group | P3_1_ | P2_1_2_1_2_1_ | C222_1_ | C222_1_ |
| Cell dimensions |  |  |  |  |
| *a*, *b*, *c* (Å) | 68.56, 68.56, 220.21 | 91.04, 105.75, 105.75 | 68.64, 85.90, 84.02 | 68.28, 85.95, 84.14 |
|    γ (°) | 90, 90, 120 | 90, 90, 90 | 90, 90, 90 | 90, 90, 90 |
| Resolution (Å) | 57.33-2.4 (2.486-2.40) | 57.78-1.63 (1.60-1.60) | 84.02-2.04 (1.99-1.99) | 53.46-1.75 (1.72-1.72) |
| *R*_merge_ | 0.195 (0.662) | 0.0208 (0.145) | 0.0270 (0.0650) | 0.092 (0.723) |
| *R*_pim_ | 0.103 (0.335) | 0.032 (0.285) | 0.048 (0.139) | 0.037 (0.334) |
| *I* / σ*I* | 5 (2) | 20.3 (4.0) | 15.2 (7.4) | 019.6 (3.7) |
| CC 1/2 | 0.959 (0.533) | 0.999 (0.947) | 0.998 (0.984) | 0.999 (0.895) |
| Total reflections | 209141 (32542) | 267786 (26294) | 34946 (3436) | 356595 (13707) |
| Unique Reflections | 45347 (6668) | 133925 (13159) | 17477 (1722) | 26016 (1280) |
| Completeness (%) | 100.0 (100.0) | 99.0 (99.0) | 100.0 (100.0) | 97.9 (93.0) |
| Redundancy | 4.6 (4.9) | 2.0 (2.0) | 3.5 (9.8) | 13.7 (10.7) |
|  |  |  |  |  |
| Refinement |  |  |  |  |
| Resolution (Å) | 57.33-2.40 | 47.29-1.60 | 38.25-1.987 | 33.06-1.72 |
| No. reflections | 45285 (4509) | 133895 (13159) | 17471 (1716) | 25986 (2457) |
| *R*_work_ / *R*_free_ | 16.1/23.1 | 18.1/20.2 | 16.1/19.3 | 17.0/20.5 |
| No. atoms | 6717 | 6875 | 1814 | 1792 |
| Protein | 6363 | 6118 | 1598 | 1558 |
| Water | 274 | 622 | 169 | 196 |
| Zn | 4 | 4 | 1 | 1 |
| *Refined B*-factors (Å^2^) |  |  |  |  |
| Overall | 32.31 | 21.57 | 19.93 | 21.59 |
| Compound | 36.42 | 35.02 | 47.47 | 33.69 |
| water | 30.26 | 28.22 | 27.70 | 29.57 |
| R.m.s. deviations |  |  |  |  |
| Bond lengths (Å) | 0.024 | 0.027 | 0.258 | 0.051 |
| Bond angles (°) | 1.07 | 1.27 | 3.40 | 0.89 |
| Ramachandran analysis |  |  |  |  |
| Favored (%) | 96.4 | 99 | 98.5 | 100 |
| Disallowed (%) | 0.1 | 0.1 | 0 | 0 |
