# Supplementary material for: A small molecule reacts with the p53 somatic mutant Y220C to rescue wild-type thermal stability
Source: Cancer Discov. Author manuscript; Available in PMC 2023 Jan 14. (PMC9827106; doi:10.1158/2159-8290.CD-22-0381)
Supplement: 2 [file NIHMS1842090-supplement-2.pdf]

Supplementary Figure 1

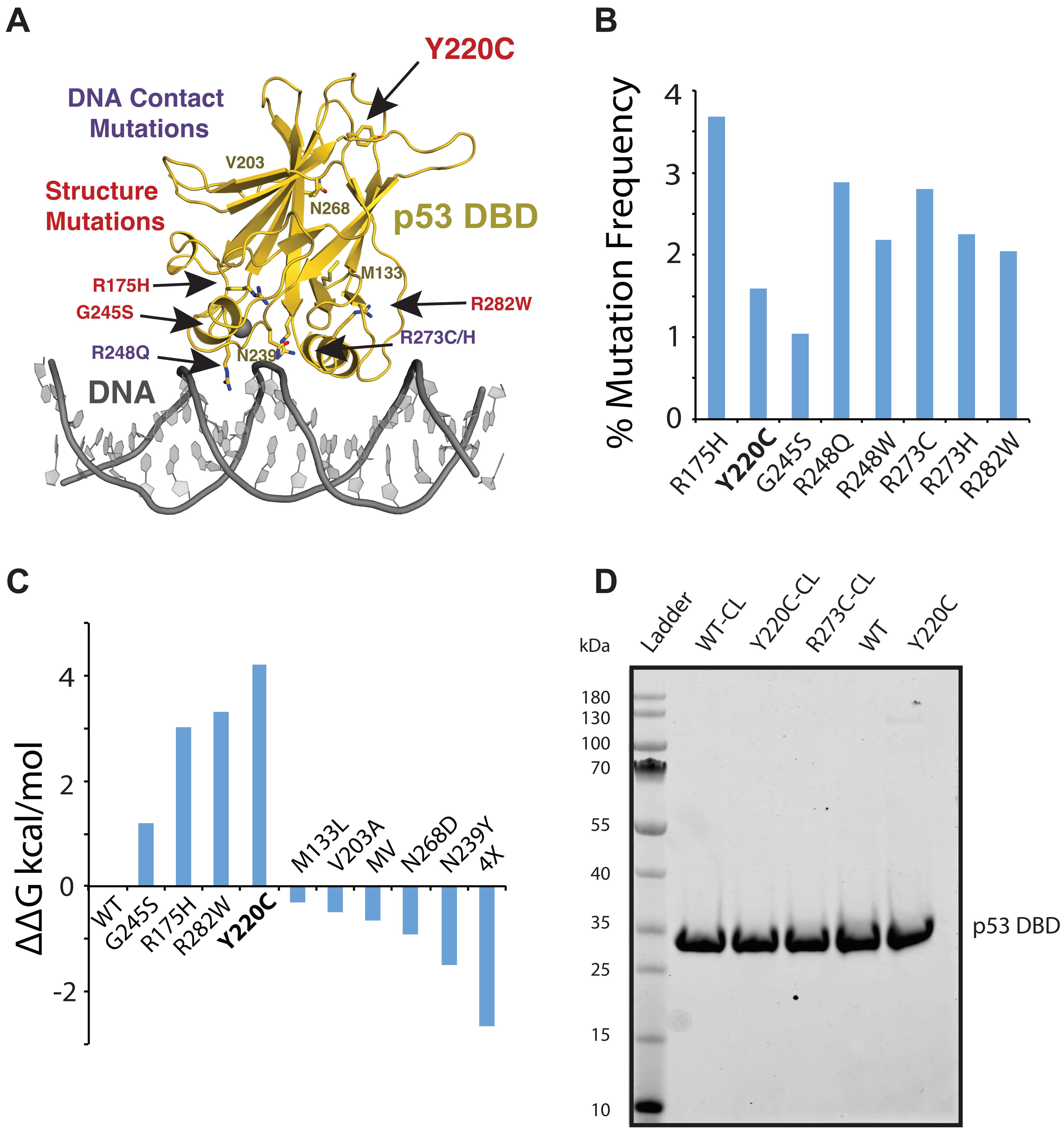

Supplementary Figure S1: **p53 hotspot mutations** (**A**) Crystal structure of p53 bound to DNA PDB 1TUP with structural and DNA-contact mutations highlighted. (**B**) Frequency of hotspot mutations out of total p53 alterations based upon data generated by the TCGA Research Network: https://www.cancer.gov/tcga. (**C**) The urea-denaturation free energy values from hotspot mutations and stabilizing mutations plotted from previous studies (7). (**D**) SDS-PAGE of recombinant protein used in study stained with Coomassie blue.
