# Supplementary material for: A small molecule reacts with the p53 somatic mutant Y220C to rescue wild-type thermal stability
Source: Cancer Discov. Author manuscript; Available in PMC 2023 Jan 14. (PMC9827106; doi:10.1158/2159-8290.CD-22-0381)
Supplement: 3 [file NIHMS1842090-supplement-3.pdf]

Supplementary Figure 2

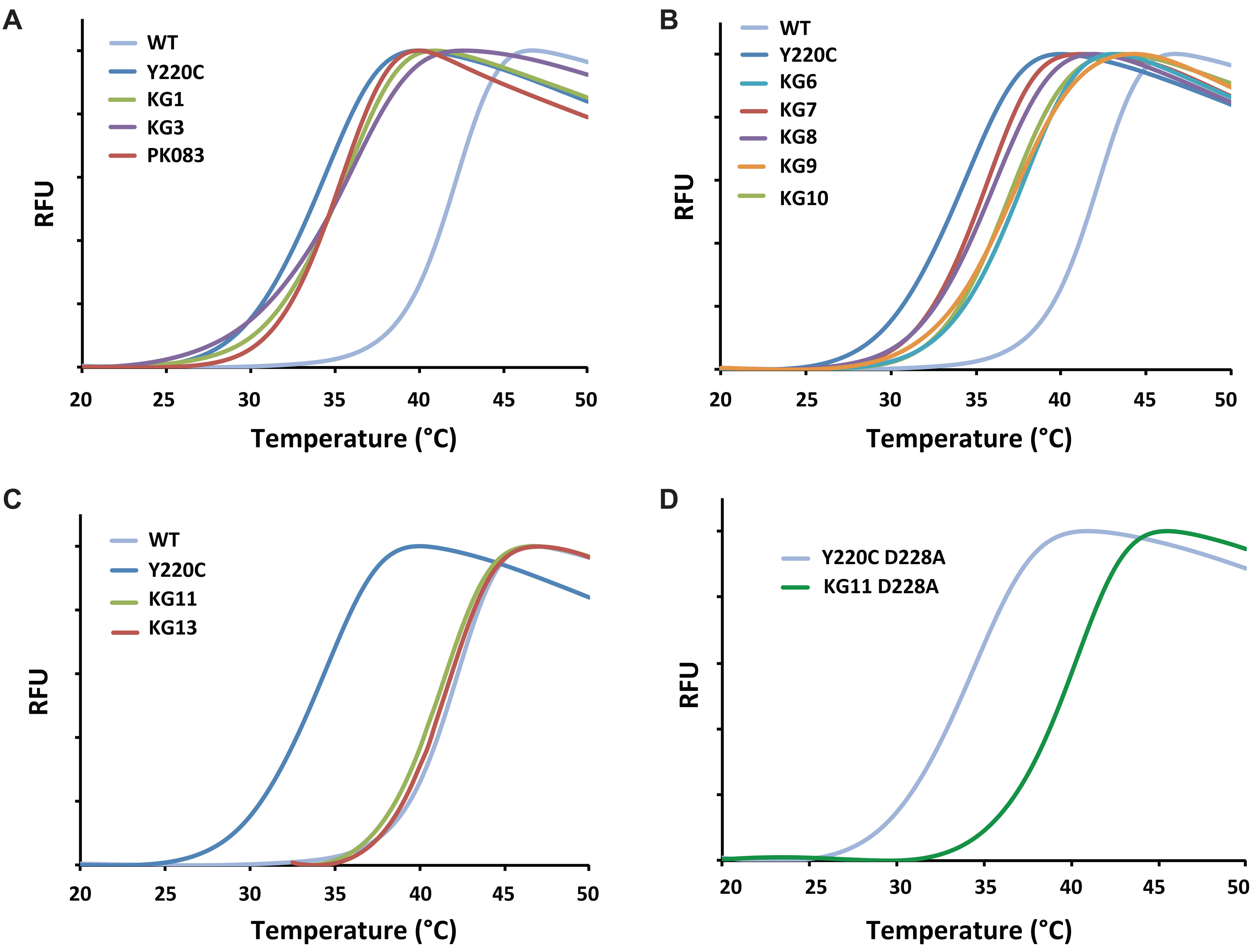

Supplementary Figure S2: **Differential scanning fluorimetry** (**A**) Carbazole series plot of average values from differential scanning fluorimetry. (**B**) Indole series plot of average values from differential scanning fluorimetry. (**C**) Azaindole series plot of average values from differential scanning fluorimetry. (**D**) p53 Y220C-CL D228A DMSO or KG11 adduct plot of average values from differential scanning fluorimetry.
