# Supplementary material for: A small molecule reacts with the p53 somatic mutant Y220C to rescue wild-type thermal stability
Source: Cancer Discov. Author manuscript; Available in PMC 2023 Jan 14. (PMC9827106; doi:10.1158/2159-8290.CD-22-0381)
Supplement: 4 [file NIHMS1842090-supplement-4.pdf]

## Supplementary Figure 3

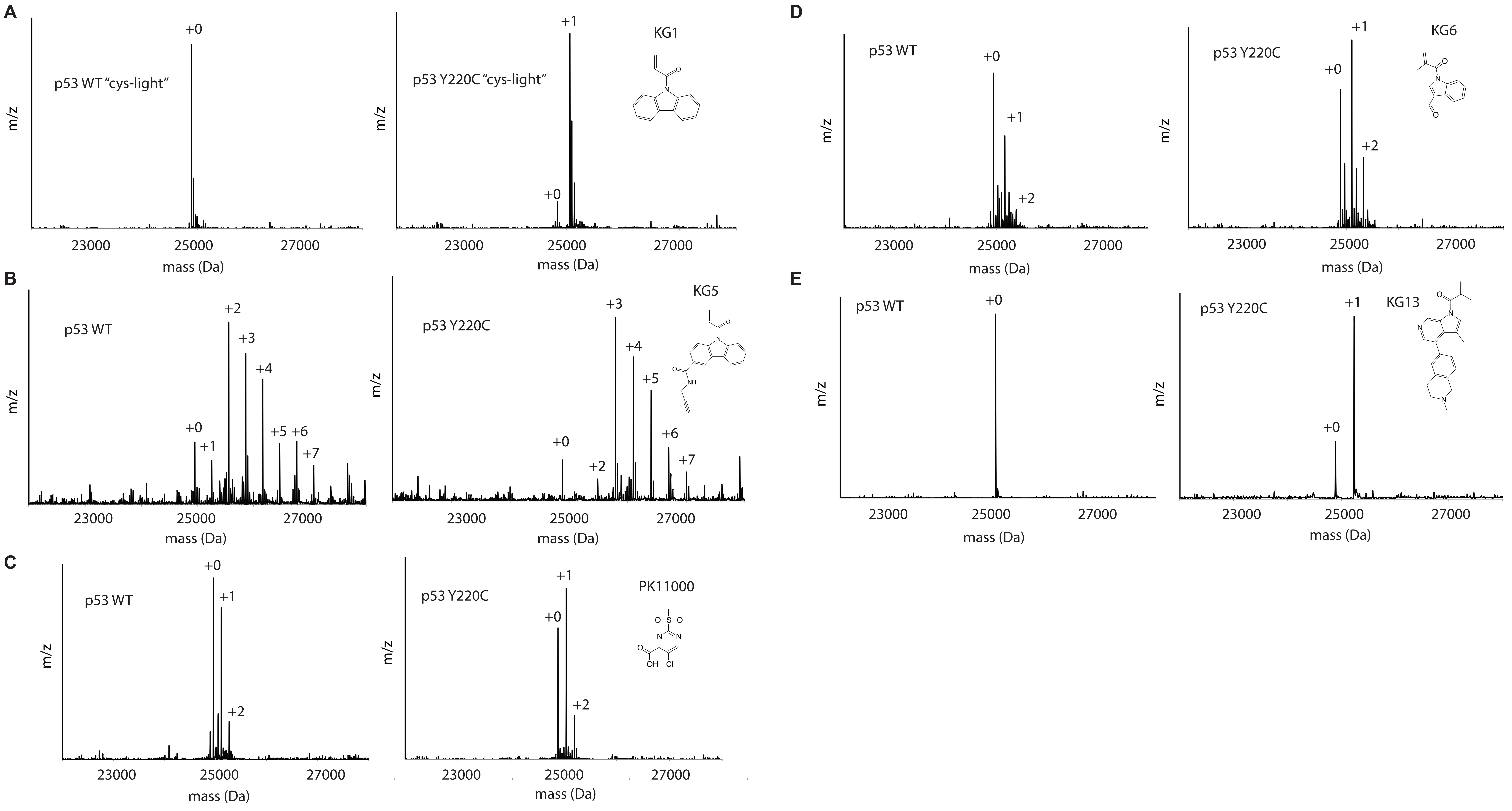

**Supplementary Figure S3: Example LC/MS spectra from carbazole and indole series** (**A**) LC/MS spectra from p53 WT-CL or p53 Y220C-CL labeling with KG1. (**B**) LC/MS spectra from p53 WT or p53 Y220C labeling with KG5. (**C**) LC/MS spectra from p53 WT or p53 Y220C labeling with PK11000. (**D**) LC/MS spectra from p53 WT or p53 Y220C labeling with KG6. (**E**) LC/MS spectra from p53 WT or p53 Y220C labeling with KG13.
