# Supplementary material for: A small molecule reacts with the p53 somatic mutant Y220C to rescue wild-type thermal stability
Source: Cancer Discov. Author manuscript; Available in PMC 2023 Jan 14. (PMC9827106; doi:10.1158/2159-8290.CD-22-0381)
Supplement: 5 [file NIHMS1842090-supplement-5.pdf]

Supplementary Figure 4

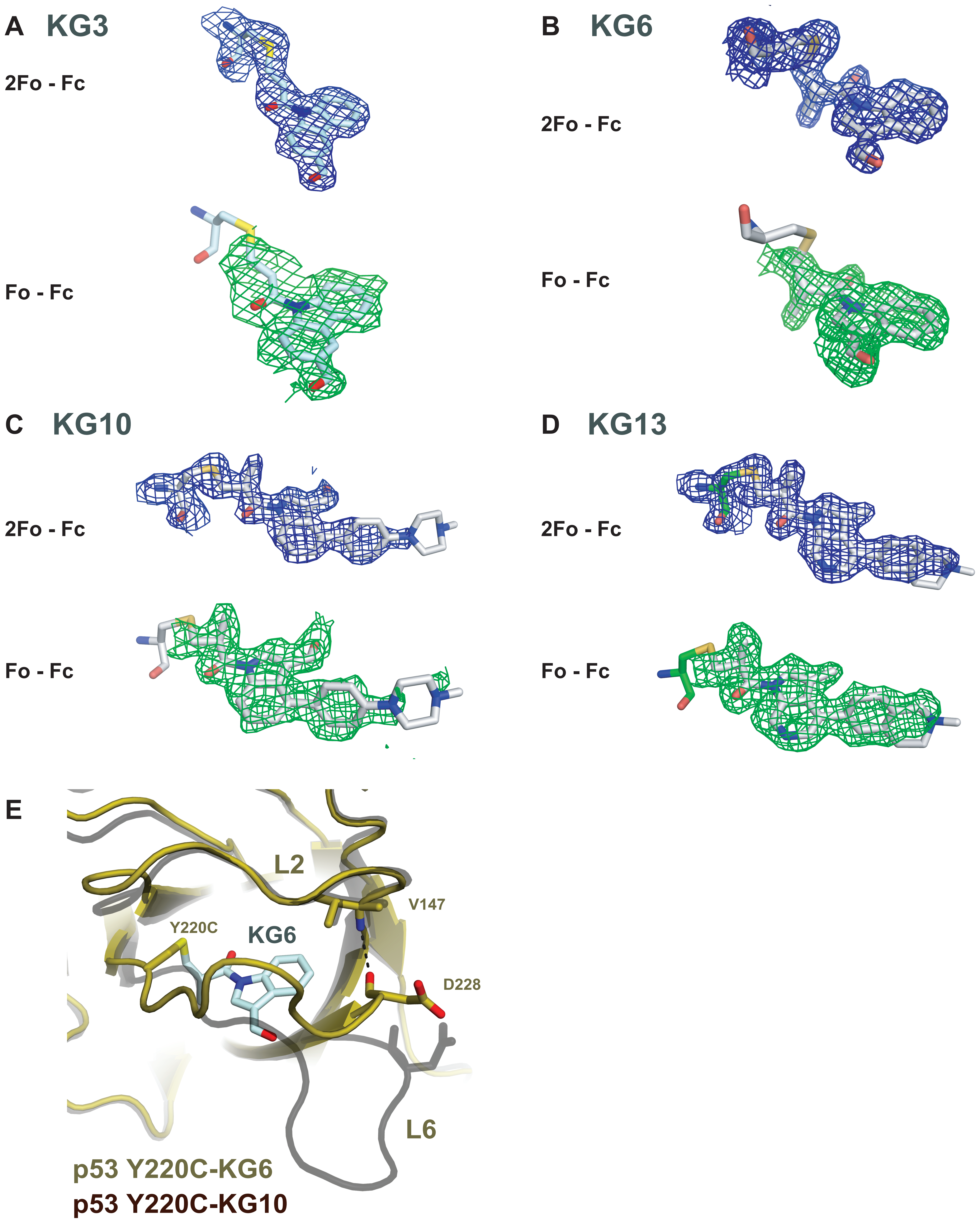

Supplementary Figure S4: Ligand electron density maps (**A**) 1σ 2Fo-Fc electron density map and 1.5σ Fo-Fc simulated annealing omit density map for KG3. (**B**) 1σ 2Fo-Fc electron density map and 1.5σ Fo-Fc simulated annealing omit density map for KG6. (**C**) 1σ 2Fo-Fc electron density map and 1.5σ Fo-Fc simulated annealing omit density map for KG10. (**D**) 1σ 2Fo-Fc electron density map and 1.5σ Fo-Fc simulated annealing omit density map for KG13. (**E**) Allosteric stabilization by KG6. D228 makes intramolecular contacts with V147 amine rather through a small molecule directly.
