# Supplementary material for: A small molecule reacts with the p53 somatic mutant Y220C to rescue wild-type thermal stability
Source: Cancer Discov. Author manuscript; Available in PMC 2023 Jan 14. (PMC9827106; doi:10.1158/2159-8290.CD-22-0381)
Supplement: Figure S6 [file NIHMS1842090-supplement-Figure_S6.pdf]

Supplementary Figure 6

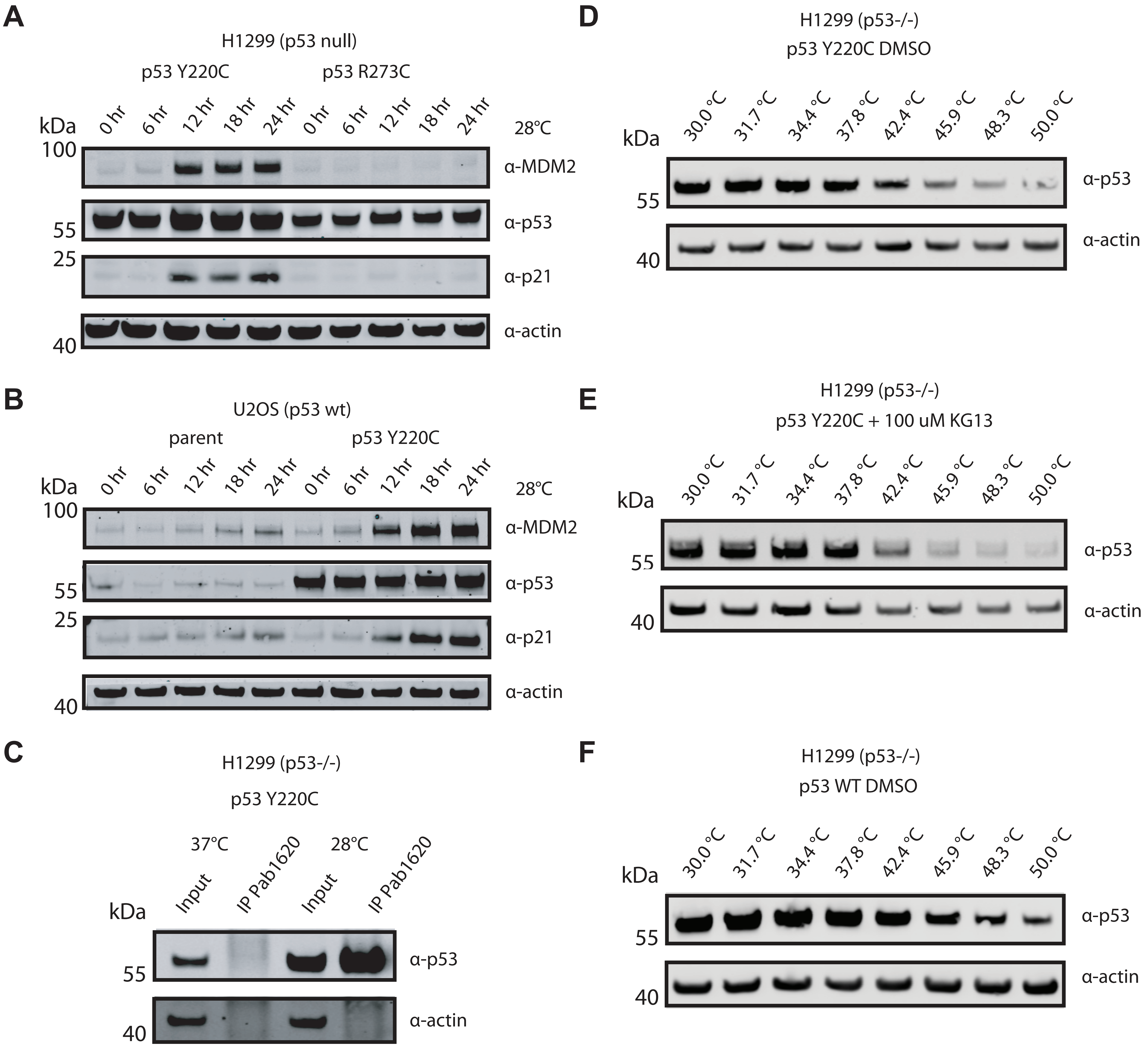

Supplementary Figure S6: Western blots and for 28°C IP and CETSA (**A**) H1299 cells stably expressing p53 Y220C or p53 R273C were transferred from 37°C to 28°C and p21/MDM2 levels were monitored by western blot at the indicated time points. (**B**) U2OS cells stably expressing p53 Y220C or parent were transferred to a 28°C incubator and p21/MDM2 levels were observed by western blot at the indicated time points. (**C**) Extracts from H1299 cells expressing p53 Y220C incubated at 37°C or 28°C for 24 hr were immunoprecipitated with the WT conformation antibody Pab1620. (**D**) CETSA western blot for p53 Y220C DMSO. (**E**) CETSA western blot for p53 Y220C treated with 100 μM KG13 for 1 hr. (**F**) CETSA western blot for p53 WT DMSO.
