# Supplementary material for: A small molecule reacts with the p53 somatic mutant Y220C to rescue wild-type thermal stability
Source: Cancer Discov. Author manuscript; Available in PMC 2023 Jan 14. (PMC9827106; doi:10.1158/2159-8290.CD-22-0381)
Supplement: Figure S7 [file NIHMS1842090-supplement-Figure_S7.pdf]

Supplementary Figure 7

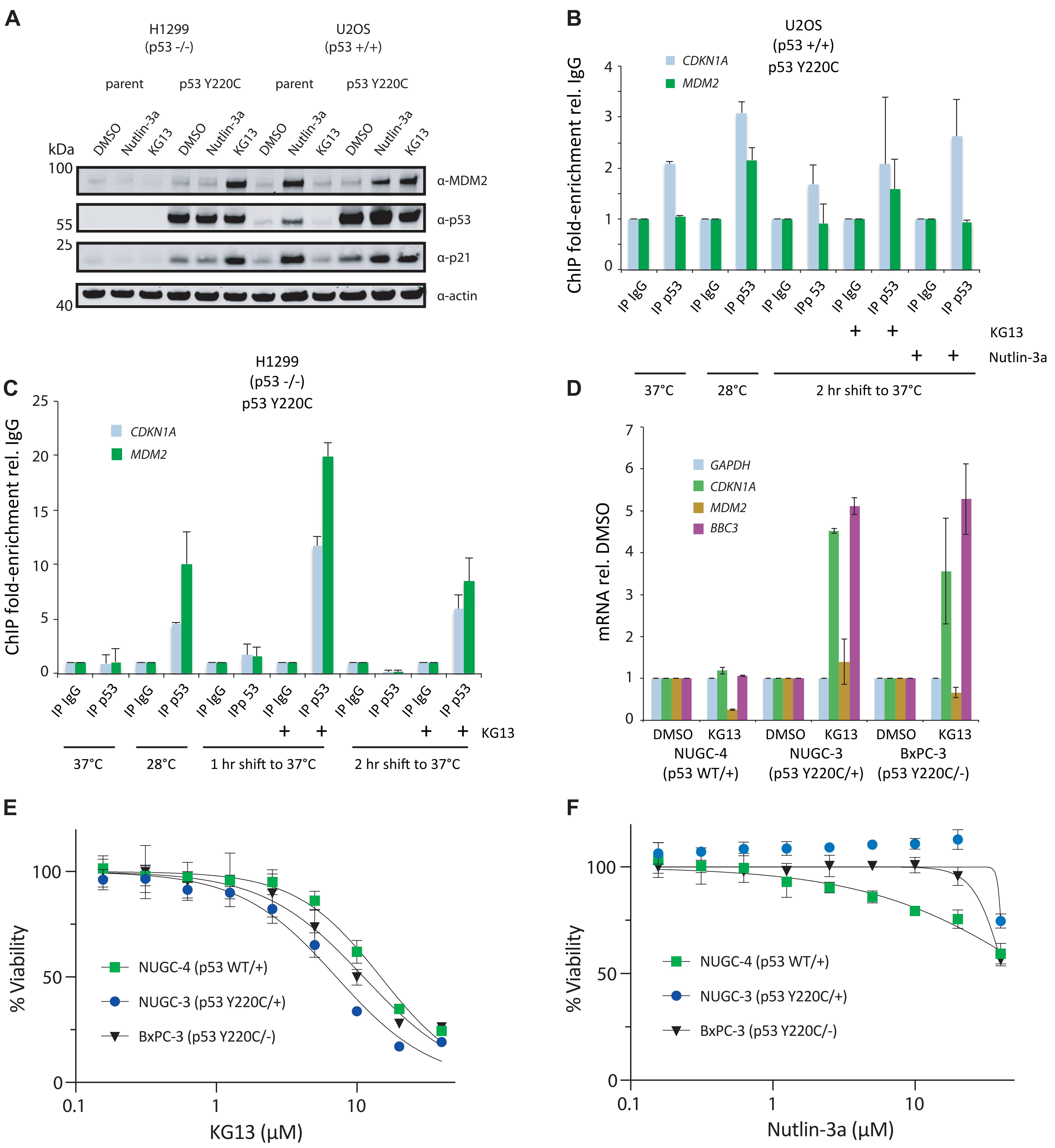

Supplementary Figure S7: Extended p53 Y220C KG13 cellular activity (**A**) Western blot for isogenic cell panel treated with 10 μM Nutlin-3a constant or 10 μM KG13 for 1 hr at 28°C, washed out, then moved to 37°C for 8hr. (**B**) ChIP p53 enrichment for U2OS cells expressing p53 Y220C. (**C**) p53 ChIP enrichment at CDKN1A and MDM2 promoters relative to IgG following KG13 treatment in H1299 cells expressing p53 Y220C. (**D**) RT-qPCR results for 25 μM KG13 treated NUGC-4, NUGC-3, and BxPC-3 cells. (**E**) Viability assay for KG13 treatment. (**F**) Viability assay for Nutlin-3a treatment.
