# Supplementary material for: A small molecule reacts with the p53 somatic mutant Y220C to rescue wild-type thermal stability
Source: Cancer Discov. Author manuscript; Available in PMC 2023 Jan 14. (PMC9827106; doi:10.1158/2159-8290.CD-22-0381)
Supplement: 6 [file NIHMS1842090-supplement-6.pdf]

Supplementary Figure 5

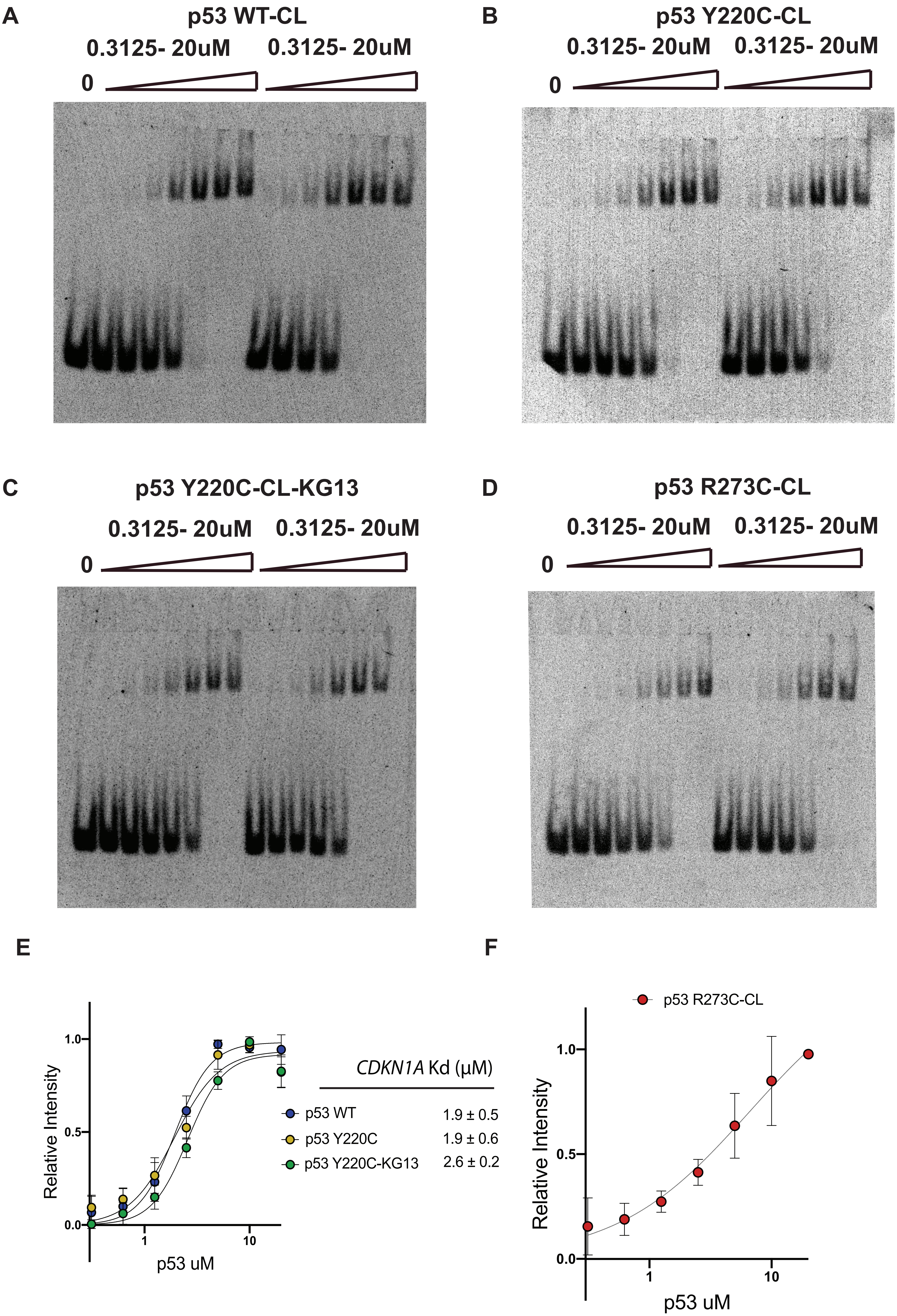

Supplementary Figure S5: ***CDKN1A*** **EMSA gels and intensity plots** (**A**) EMSA for p53 WT-CL. (**B**) EMSA for p53 Y220C-CL. (**C**) EMSA for p53 Y220C-CL complexed with KG13. (**D**) EMSA for p53 R273C-CL. (**E**) Upper band intensities plotted for each protein construct. (**F**) Upper band intensities plotted for p53 R273C-CL.
