# Supplementary material for: A Small Molecule Reacts with the p53 Somatic Mutant Y220C to Rescue Wild-type Thermal Stability
Source: Cancer Discov. 2022 Oct 5;13(1):56–69. doi: 10.1158/2159-8290.CD-22-0381 (PMC9827106; doi:10.1158/2159-8290.CD-22-0381)
Supplement: Supplementary file 1 [file cd-22-0381_supplementary_figure_6_suppsf6.pdf]

Supplementary Figure 6

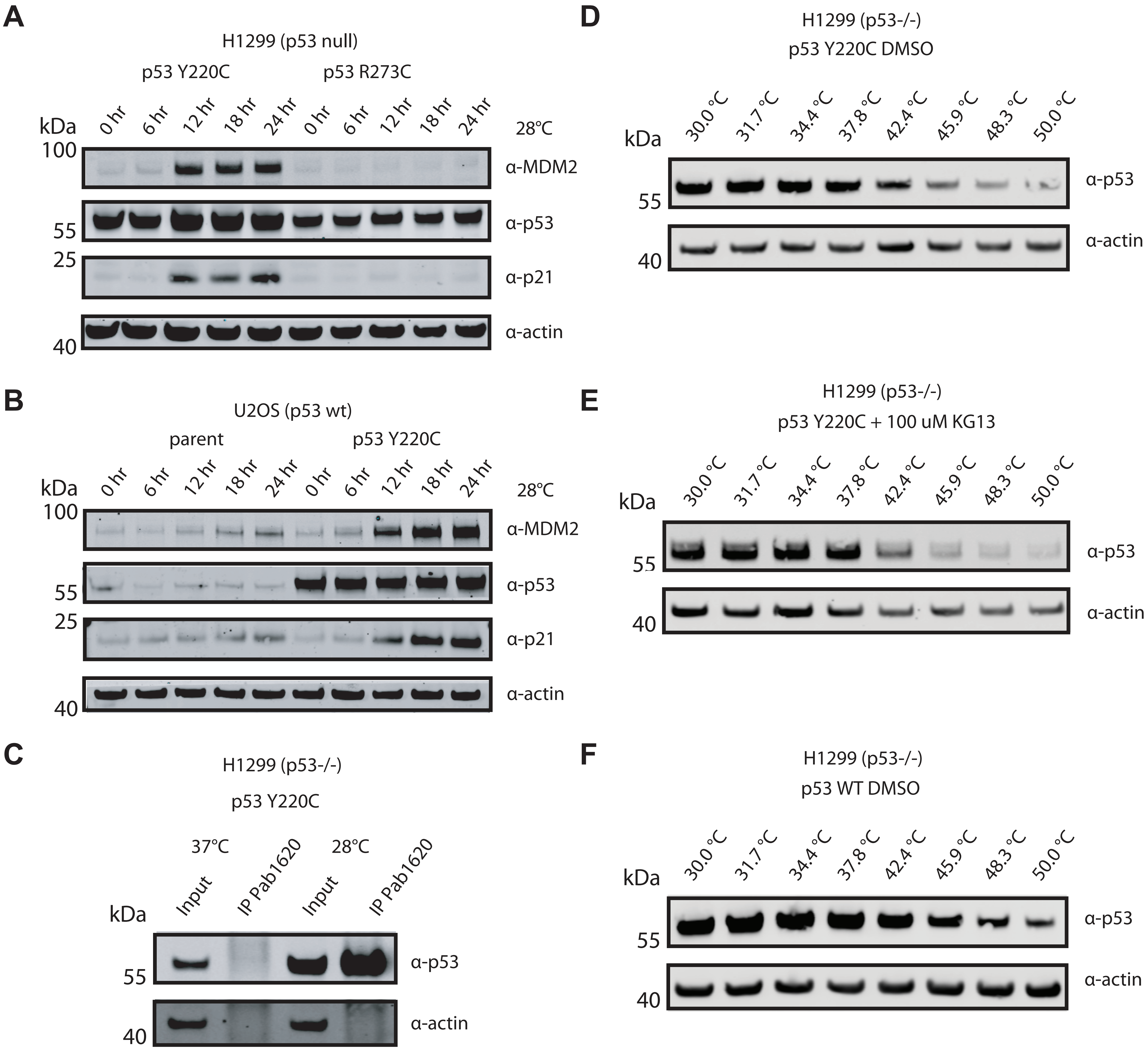

Supplementary Figure S5: ***CDKN1A*** **EMSA gels and intensity plots** (**A**) EMSA for p53 WT-CL. (**B**) EMSA for p53 Y220C-CL. (**C**) EMSA for p53 Y220C-CL complexed with KG13. (**D**) EMSA for p53 R273C-CL. (**E**) Upper band intensities plotted for each protein construct. (**F**) Upper band intensities plotted for p53 R273C-CL.
