# Supplementary material for: A Small Molecule Reacts with the p53 Somatic Mutant Y220C to Rescue Wild-type Thermal Stability
Source: Cancer Discov. 2022 Oct 5;13(1):56–69. doi: 10.1158/2159-8290.CD-22-0381 (PMC9827106; doi:10.1158/2159-8290.CD-22-0381)
Supplement: Supplementary file 2 [file cd-22-0381_supplementary_figure_7_suppsf7.pdf]

Supplementary Figure 7

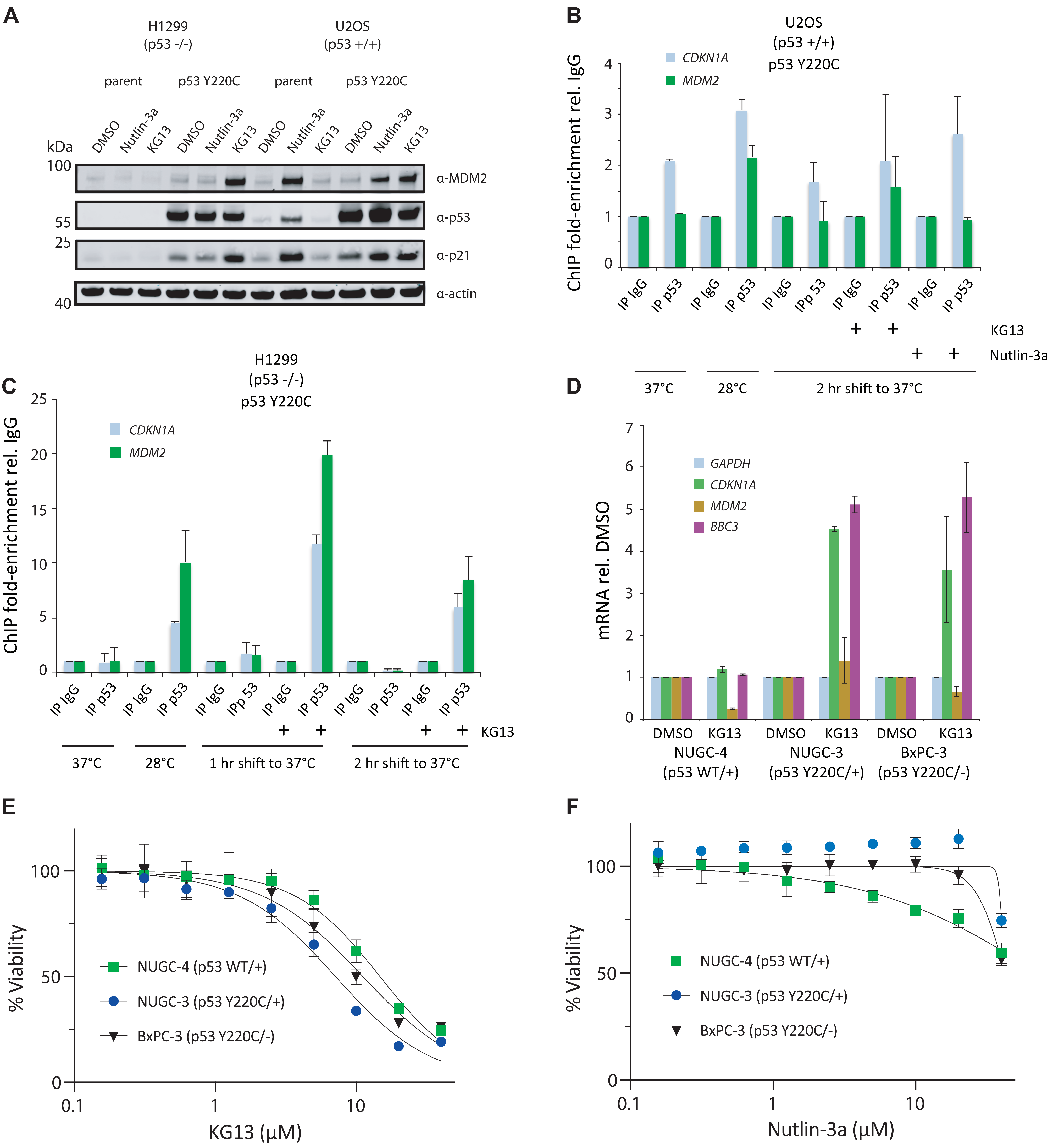

Supplementary Figure S7: Extended p53 Y220C KG13 cellular activity (**A**) Western blot for isogenic cell panel treated with 10 μM Nutlin-3a constant or 10 μM KG13 for 1 hr at 28°C, washed out, then moved to 37°C for 8hr. (**B**) ChIP p53 enrichment for U2OS cells expressing p53 Y220C. (**C**) p53 ChIP enrichment at *CDKN1A* and *MDM2* promoters relative to IgG following KG13 treatment in H1299 cells expressing p53 Y220C. (**D**) RT-qPCR results for 25 μM KG13 treated NUGC-4, NUGC-3, and BxPC-3ß cells. (**E**) Viability assay for KG13 treatment. (**F**) Viability assay for Nutlin-3a treatment.
